# Supplementary material for: Dithiine-linked metalphthalocyanine framework with undulated layers for highly efficient and stable H2O2 electroproduction
Source: Nat Commun. 2024 Jan 23;15:678. doi: 10.1038/s41467-024-44899-8 (PMC10805717; doi:10.1038/s41467-024-44899-8)
Supplement: Supplementary file 1 — Supplementary Information [file 41467_2024_44899_MOESM1_ESM.pdf]

Supplementary Information for

Dithiine-linked Metalphthalocyanine Framework with Undulated Layers for Highly Efficient and Stable H_2O_2 Electroproduction

Qianjun Zhi,[a] *Rong Jiang,*[a] *Xiya Yang,*[a] *Yucheng Jin,*[a] *Dongdong Qi,*[a] *Kang Wang,**[a] *Yunpeng Liu,**[b] *and Jianzhuang Jiang**[a]

[a] Beijing Advanced Innovation Center for Materials Genome Engineering, Beijing Key Laboratory for Science and Application of Functional Molecular and Crystalline Materials, Department of Chemistry and Chemical Engineering, School of Chemistry and Biological Engineering, University of Science and Technology Beijing, Beijing 100083, China

E-mail: kangwang@ustb.edu.cn (K.W.), jianzhuang@ustb.edu.cn (J.J.)

[b] Beijing Synchrotron Radiation Facility, Institute of High Energy Physics, Chinese Academy of Science, Beijing 100049, China

E-mail: liuyunpeng@ihep.ac.cn (Y.L.)

Table of contents

1. Experimental section.. 3
1.1 Chemicals 3
1.2 Synthesis of CoPc-O-COF-S20% …..3
1.3 Synthesis of CoPc-O 3
1.4 Synthesis of CoPc-S ….. 3
1.5 Synthesis of model molecules 4
1.6 Characterization…..4
1.7 The typical three-electrode system…..5
1.8 Preparation of the working electrode for H-cell……………………………..5
1.9 Cyclic voltammetry (CV) measurement ……….…….……….……………....5
1.10 Linear sweep voltammetry (LSV) measurement…………………………….. 6
1.11 Rotating ring-disk electrode (RRDE) measurement…………………….....6
1.12 Koutecky-Levich (K-L) plot measurement……………………………………6
1.13 Double-layer capacitances (C_{dl})……………………………………………..….7
1.14Determination of surface concentration of electroactive cobalt phthalocyanine units in CoPc-O-COF and CoPc-S-COF…………………………....7
1.15 Other electrochemical measurement………………………………………..... 8
1.16 iR-Correction………………………………………………………………………8
1.17 H_2O_2 yield in the flow cell…………………………………………………………8
1.18 Calculation details…………………………………………………..…..... 10
2. Supplementary Figures and Tables...13
3. Supplementary References..44

1. Experimental section

1.1 Chemicals. All the starting materials were purchased from TCI or Admas and used without further purification. Cobalt(II) 1,2,3,4,8,9,10,11,15,16,17,18,22,23,24,25-hexadecafluoro-29H,31H-phthalocyanine ($CoPcF_{16}$) and H_2PcF_{16} were synthesized according to literature methods.[1,2] N,N-Dimethylformamide anhydrous (DMF), N,N-dimethyl acetamide (DMAc), p-xylene, triethylamine, acetone, dichloromethane, tetrahydrofuran, and methanol were purchased from Admas Reagent. 1,2,4,5-Tetrahydroxybenzene (THB) was purchased from TCI. 1,2,4,5-Benzenetetrathiol (BTT) was synthesized according to literature methods.[3]

1.2 Synthesis of CoPc-O-COF-S 20%. CoPc-O-COF-S 20% was prepared by following the synthesis procedure of CoPc-O-COF with 0.016 mmol THB and 0.004 mmol BTT instead of 0.02 mmol THB as starting material.

1.3 Synthesis of CoPc-O. $CoPcF_{16}$ (86 mg, 0.1 mmol), benzene-1,2-dyhydroxy (44 mg, 0.4 mmol), and potassium carbonate (1 mmol, 138 mg) were added into dry DMF. The reaction mixture was then heated at 80 °C for 24 h. After cooling down, 20 ml 1 M HCl was added into the reaction mixture. The crude product was isolated by filtration and then solvent exchanged with ethanol, acetone, and chloroform to wash out all impurities and unreacted starting materials, giving CoPc-O in a yield of 85%, Supplementary Fig. 50. FT-IR (neat, cm^{-1}): 1614, 1460, 1402, 1306, 1269, 1184, 1086, 795, 617; High resolution MS (m/z): $[M+H]^+$ calcd for $C_{56}H_{17}N_8O_8Co$, 1140.7; found 1140.2.

1.4 Synthesis of CoPc-S. CoPc-S was prepared by following the synthesis procedure of CoPc-O with 0.4 mmol benzene-1,2-dithiol instead of 0.04 mmol benzene-1,2-dyhydroxy as the starting material. Yield: 95%, Supplementary Fig. 50; FT-IR (neat, cm^{-1}): 1618, 1461, 1261, 1086, 797, 712, 690, 613; High resolution MS

(m/z): $[M+H]^+$ calcd for $C_{56}H_{17}N_8S_8Co$, 1269.2; found 1269.9.

1.5 Synthesis of model molecules.

Model molecule 1. 1, 2-Difluorobenzene (114 mg, 0.1 mmol), 1, 2-benzenedithiol (142 mg, 0.1 mmol), and potassium carbonate (1 mmol, 138 mg) were added into dry DMF. The reaction mixture was then heated at 80 °C for 24 h. After cooling down, 20 ml 1 M HCl was added into the reaction mixture. The light yellow powder was isolated by filtration and then washed with ethanol and acetone with a yield of 80%. The obtained crude powder was recrystallized with DMF/methanol to give model molecule 1 as colorless crystal.

Model molecule 2. Model molecule **2** was prepared by following the synthesis procedure of model molecule **1** with 1, 2, 4, 5-tetrachlorobenzene (0.05 mmol) instead of 1, 2-difluorobenzene (0.1 mmol) as the starting material with a yield of 88%. The obtained crude powder was recrystallized by temperature-gradient vacuum sublimation to give model molecule **2** as colorless crystal.

1.6 Characterization. Powder X-ray diffraction (PXRD) analyses were performed using a PANalytical Empyrean diffractometer operating at 45 kV voltage and 40 mA current with Cu-Kα X-ray radiation (λ = 0.154056 nm). Fourier transform infrared (FT-IR) spectra were recorded by a Bruker Tensor 37 infrared spectrometer. Raman spectra were recorded on a Horiba LabRAM HR Evolution spectrometer. The solid-state ^{13}C NMR spectra were recorded on a 400 MHz WB Solid-State NMR Spectrometer. Scanning electron microscopy (SEM) images were obtained by a SU8010 electron microscopy. Transmission electron microscopy (TEM) images were collected from a HT7700 electron microscope at 100 kV. High-resolution TEM (HRTEM) and energy dispersive spectroscopy (EDS) mapping images were collected by transmission electron microscopy (JEM-2200FS) at an operation voltage of 200 kV. Thermogravimetric analysis (TGA) was characterized by SDTA851e thermo-analyzer. The X-ray Photoelectron spectroscopy (XPS) was acquired using an EscaLab Xi+ spectrometer. N_2 adsorption-desorption isotherms were measured on a ASAP 2020

PLUS HD88 apparatus at 77 K and the surface areas were calculated by the Brunauere Emmette Teller (BET) method. Inductively coupled plasma-optical emission spectroscopy (ICP-OES) was performed on an Agilent 725ES & Agilent 5110. Electrochemical measurements were conducted on the CHI 760E workstation (CH Instruments, Inc.) with a RRDE-3A rotator by employing a typical three-electrode system (the corresponding methods were specified below). The XAFS spectra at Co K-edge were acquired at the XRD station of beamline 4B9A of BSRF in the Beijing Synchrotron Radiation Facility. The acquired EXAFS raw data were then background-subtracted, normalized, and Fourier-transformed by using the ATHENA program in the IFEFFIT software package. Least-squares curve parameter fitting was performed using the ARTEMIS module of IFEFFIT software packages.

1.7 The typical three-electrode system. A typical three-electrode system was employed, using a commercial glassy carbon (GC) electrode (4 mm diameter, 0.1256 cm^2), a platinum wire, and Ag/AgCl electrode (in saturated KCl solution) with salt bridge as the working, counter, and reference electrodes, respectively. All potentials were referred to the reversible hydrogen electrode by adding a value of (0.198 + 0.0592 × pH) V. All the electrochemical tests in this study were conducted at least three times to ensure the accuracy of the measurement. Besides, the iR correction was applied to get rid of the influence of the Ohmic resistance.

1.8 Preparation of the working electrode for H-cell. The catalyst-modified working electrode was fabricated by casting an appropriate amount of catalyst ink, which was obtained by ultrasonically dispersing the catalyst (4 mg) into 1.0 mL ethanol containing 20 μL 5wt% Nafion. Then, 5 μL of the mixture was dropped onto a polished glassy carbon electrode (4 mm in diameter). The loaded electrode was placed in a 60 °C oven for 10 min to dry and then was taken out to cool down before all the tests. The loading amount of each catalyst was kept at 0.16 mg cm^{-2}.

1.9 Cyclic voltammetry (CV) measurement. Prior to the test, the electrolyte (0.1 M

KOH solution) was bubbled with O_2 for at least 30 min to make it saturated with O_2, and a constant oxygen flow was kept during the measurement. The data were recorded at the scan rate of 100 mV s^{-1} under static conditions when the system became stable. The resulting data were corrected to remove the iR drop. Before the ORR, the pre-activation process by scanning CV curves (40 cycles, scan rate: 100 mV/s) was performed on RRDE to electrochemically clean it until stable CV curves were obtained.

1.10 Linear sweep voltammetry (LSV) measurement. The electrochemical clean process was carried out through scanning CV curves for 20 cycles (potential range: 1.1-0.05 V, scanning rate: 0.5 V/s) before LSV measurement. The rotating speed of the working electrode is increased from 400 to 1600 rpm at the scan rate of 5 mV s^{-1}. The resulting data were corrected to remove the iR drop and the double-layer effect.

1.11 Rotating ring-disk electrode (RRDE) measurement. The rotating speed of the working electrode was fixed at 1600 rpm with the scan rate of 10 mV s^{-1} for the RRDE test. Pt ring potential was maintained at 1.2 V vs. RHE to detect the H_2O_2 generated. The electron transfer number (n) is calculated *via* the following equation.

$$n = 4I_d / (I_d + I_r / N) \qquad (1)$$

$$H_2O_2\% = 200*(I_r / N) / (I_d + I_r / N) \qquad (2)$$

Where I_d stands for the disk current, I_r represents the ring current, and N is the current collection efficiency of the Pt ring. The collection efficiency (N) on the RRDE electrode was determined in 1 M KCl + 10 mM $K_3[Fe(CN)_6]$ at different rotate speeds, in which the potential on disk was scanned from 0.5 to -0.1 V vs. Ag/AgCl to reduce Fe^{3+} to Fe^{2+} while the potential on Pt ring was held at 0.5 V vs. Ag/AgCl to oxidize Fe^{2+} to Fe^{3+}, Supplementary Fig. 51.[4]

1.12 Koutecky-Levich (K-L) plot measurement. The working electrode was scanned cathodically at the rate of 10 mV s^{-1} with the rotation speed from 400 to 2500 rpm. Koutecky-Levich (K-L) plots (J^{-1} vs $\omega^{-1/2}$) were analyzed at 0.3-0.6 V *via* the

following equation:

$$1/J = 1/J_L + 1/J_K = 1/(B\omega^{1/2}) + 1/J_K \quad (3)$$

$$B = 0.2nFC_0D_0^{2/3}\,v^{-1/6} \quad (4)$$

$$J_K = nFkC_0 \quad (5)$$

Where J is the measured current density, J_K and J_L are the kinetic and limiting current densities, ω is the angular velocity, n is transferred electron number, F (96485 C mol^{-1}) is the Faraday constant, D_0 is the diffusion coefficient of O_2 in 0.1 M KOH (1.9×10^{-5} cm^2 s^{-1}), C_0 is the bulk concentration of O_2 (1.26×10^{-3} mol L^{-1}), v is the kinetic viscosity of the electrolyte (0.01 cm^2 s^{-1}), and k is the electron-transfer rate constant. The constant 0.2 is adopted when the rotation speed is expressed in rpm.

1.13 Double-layer capacitances (C_{dl}). The double-layer capacitances (C_{dl}) of different catalysts were calculated by recording different CV curves at a variety of sweep rates of 2, 4, 6, 8, and 10 mV s^{-1} in the potential range of 0.89-0.99 V vs. RHE. Within the range of the potential where no electrochemical reaction occurs, the limiting current density (J) and scanning rate (v) present the following functional relationship:

$$J = vC_{dl} \quad (6)$$

1.14 Determination of surface concentration of electroactive cobalt phthalocyanine units in CoPc-O-COF and CoPc-S-COF. To estimate the surface concentration of electroactive cobalt phthalocyanine units in COFs, CV tests of CoPc-O-COF and CoPc-S-COF in CO_2 saturated 0.1 M KOH on the scan rate between 20 to 100 mV s^{-1} were conducted. The peak current shows a linear dependence on the scan rate. Calculation of surface coverage (τ_0) for both COFs: Regression of the linear regime between 20 and 100 mV s^{-1} with equation:

$$\text{Slope} = n^2F^2A\tau_0/4RT \quad (7)$$

where n is the number of electrons involved, F is the Faraday constant, A is geometrical surface area of the electrode (3.14×0.15×0.15 cm^2), τ_0 is the surface coverage, R is the gas constant, T is test temperature (298 K).

1.15 Other electrochemical measurement: For the chronoamperometry measurement, the potential was fixed at 0.52 V vs. RHE. The 0.1 M KOH containing 1 mM KSCN and 10 mM H_2O_2 was used as the electrolyte for the poisoning test and H_2O_2 reduction reaction measurements, respectively. Electrochemical impedance spectroscopy (EIS) measurements were tested in a quartz cell filled with 0.2 M Na_2SO_4 electrolyte. The Na_2SO_4 electrolyte was purged with N_2 for 1 h prior to the measurements. The resistance value (R) of the COF can be obtained based on the drain current and voltage curves, and the electronic conductivity (σ) can be determined according to the following equation:

$$\sigma = 1/\rho = L/SR = L/\pi r^2 R \qquad (8)$$

Where ρ and σ are the electrical resistivity and electronic conductivity, respectively. And R, S, L, and r represent the resistance value, area, thickness, and radius of the COF pellet, respectively.

1.16 iR-Correction. The iR correction has been adopted to remove the influence of Ohmic resistance on the ORR measurements. Specifically, the electrochemical alternating current EIS was utilized to measure the Ohmic resistance under the ORR conditions. The potentials were calculated *via* the following equation.

$$E_{\text{iR-corrected}} = E - IrV \qquad (9)$$

Where i is the current, R is the uncompensated ohmic electrolyte resistance measured *via* high frequency A.C. impedance in O_2-saturated 0.1 M KOH solution, which is around 50 Ω for all the tested samples.

1.17 H_2O_2 yield in the flow cell. The electro-synthesis of H_2O_2 through ORR was carried out using a flow cell setup in a two-compartment cell separated by Nafion membrane. The ink of CoPc-O-COF catalyst was dropped on the carbon paper (actual working area: 0.25 cm^2, catalyst loading: 0.16 mg cm^{-2}) and was assembled into the gas diffusion layer to acquire the cathode. The Ag/AgCl electrode with a salt bridge and Pt sheet were employed as the reference electrode and anode, respectively. In the

flow cell test, the electrolyte (1 M KOH, 80 ml) was recycled across each compartment at the flow speed of 12 mL min^{-1} and the O_2 supply rate was maintained at 20 mL min^{-1} flowing through the cathode. For the test of H_2O_2 production rate and stable H_2O_2 production, the cathode current density was maintained at -125 mA cm^{-2}. In addition, the flow cell tests in the neutral electrolyte (1 M Na_2SO_4, 20 ml) were also carried out under the same condition. For the corresponding test of H_2O_2 production rate and stable H_2O_2 production in the neutral electrolyte, the cathode current density was maintained at -100 mA cm^{-2}. The overpotential can be calculated by the following equation[5]:

$$\eta = U_0 - U \qquad (10)$$

Where η is the overpotential, U is the applied electrode potential, and U_0 is the standard equilibrium potential of 0.76 V *versus* RHE at 298 K in an alkaline electrolyte.[6]

The faradaic efficiency of the H_2O_2 production (FE_{H2O2}) was determined by the following formula:

$$FE_{H2O2}\,(\%) = 2FCV/Q \qquad (11)$$

Where F indicates the faraday constant (96485 C mol^{-1}), C is the produced H_2O_2 concentration (mol L^{-1}) in the electrolyte, *V* refers to the volume of electrolyte (L), Q represents the consumed charge amount (C). In this chronoamperometry process, the value of Q was calculated according to the following formula:

$$Q = It \qquad (12)$$

Where *I* indicates the constant current and the *t* refers to running time.

Detection of hydrogen peroxide concentration: The concentration of H_2O_2 was determined by UV-vis spectrophotometer. [7] The UV spectrophotometry $Ce(SO_4)_2$ color method followed the reaction equation (13), in which the yellow Ce^{4+} reacted with H_2O_2 to generate achromatous Ce^{3+}.

$$2Ce^{4+} + H_2O_2 \rightarrow 2Ce^{3+} + 2H^{+} + O_2 \qquad (13)$$

The concentration of produced H_2O_2 can be calculated by the following formula:

$$c(H_2O_2) = ½ \times c(Ce^{4+}) \quad (14)$$

The yellow transparent $Ce(SO_4)_2$ solution (1 mM) was prepared by dissolving 16.6 mg $Ce(SO_4)_2$ in 50 mL 0.5 M sulfuric acid solution. To obtain the calibration curve, H_2O_2 with known concentration was added to $Ce(SO_4)_2$ solution and measured by a UV/vis spectrometer, Supplementary Fig. 46. Based on the linear relationship between the signal intensity and Ce^{4+} concentration, the H_2O_2 concentrations of the samples could be obtained.

1.18 Calculation details. The combining energies between O_2 and different catalytic sites was investigated by density functional theory (DFT) calculation at the level of M06-L-D3/6-311G(d)/SDD[8-12] using Gaussian 16 B.01 software.[13] According to the calculation results, Co atom is the unique reasonable catalytic site in CoPc-O-COF and CoPc-S-COF, Supplementary Table 6 and Fig. 43. When the O_2 molecule is attracted onto the central Co sites in CoPc-O-COF and CoPc-S-COF, the combining energy is ca. -0.3 eV, which shows a proper connection strength between the oxygen atom and catalytic site. In contrast, when the O_2 molecule is attracted onto the meso-N sites, the combining energy will be less than -0.05 eV, which is too weak to resist the thermal vibration at the room temperature. For the O/S/F atoms with intense negative charges, they have no chance to perform as the catalytic sites. In the calculation, once we tried to connect the O_2 molecule and O/S/F atoms, the software immediately separated them far away to > 4 Å during the optimizing process, indicating the repulsive force between them. In order to visualize the uniform conjugated system of both COFs, the localized orbital locator for π electrons (π-LOL)[14] calculation is carried out using ωB97X-D functional[15,16], Supplementary Fig. 52. As can be found, the S atoms have completely fused into the uniform conjugated system using the π-bonding in the manner of π(PcCo)-p(S)-π(Ph). Follow this long-range π linking pathway, the charge transfer from S to Co is quite smooth due to the long-range conjugated effect. This is also the case for CoPc-O-COF.

In an attempt to evaluate the $2e^-$ ORR activity of CoPc-O-COF and CoPc-S-COF, first-principles calculations were carried out via Vienna ab initio

package (VASP)[17,18] program by using projected augmented wave (PAW) potential[19] with a cutoff energy of 400 eV. The Perder-Burke-Ernzergof (PBE) functional was used within the formulation of generalized gradient approximation (GGA)[20,21] for the exchange-correlation term. The reciprocal space was sampled by the gamma point in the Brillouin zone with a grid of 2 × 2 × 1. The energy difference of 1.0×10^{-5} eV per atom and the max force of 0.02 eV/Å were set as the convergence criteria for geometry optimization. The Gibbs free energy differences ($\triangle G$) for the $2e^-$ ORR and $4e^-$ ORR processes on Co active sites in CoPc were calculated by using density functional theory (DFT) calculations carried on a Materials Studio 2017 package.[22] The structures of intermediates were optimized by Dmol3 module, following by the frequency calculation to obtain zero point vibration energy and entropy. The GGA with the PBE function and Tkatchenko-Scheffler (TS) for Density functional theory-Dispersion interactions (DFT-D) correction were employed. The convergence tolerance of energy, force, and displacement convergence were set as 1×10^{-5} Ha, 2×10^{-3} Ha, and 5×10^{-3} Å, respectively. The electron treatment was performed by double numerical plus d-functions (DNP) basis set. Bader charge analysis were carried out on the Vienna ab initio simulation package known as VASP. For the electron-electron interaction, the Perdew-Burke-Ernzerhof (PBE) functional was utilized, and the projector augmented wave (PAW) scheme was used to describe the interaction between ions and valence electrons. An energy cutoff of 500 eV was used and a k-point sampling set of 2 × 2 × 1 was allowed to relax all atomic coordinates and 4 × 4 × 1 for PDOS calculations. The periodical unit cell was surrounded by the vacuum space, which was specified to be ~30 Å. The convergence criterion for the electronic self-consistent field (SCF) loop was set to 1×10^{-6} eV/atom. The atomic structures were optimized until the residual forces of each atom were below 0.02 eV $Å^{-1}$. The atomic structures were analyzed by using the VESTA code.[23] The natural population analysis (NPA)[24] charge distribution was also calculated at the level of ωB97X-D/SDD(for Co)/6-311+G(d,p) using Gaussian 16 B.01 program.[12,13,15] According to the results of NPA calculation, the atomic charge of the inner N atoms are enhanced from $-320 \times |10^{-3}e|$ to $-373 \times |10^{-3}e|$ when the O atoms in CoPc-O-COF

are replaced by S atoms in CoPc-S-COF, Supplementary Table 7 and Fig. 53. Due to the increased negative charge located at the inner N atoms, the total repulsive force between O_a-O_b in *OOH and inner-N_4 framework in Pc macrocycle is as high as 0.076 e^2 Bohr^{-2} in CoPc-S-COF, which is obviously larger than that in CoPc-O-COF of 0.057 e^2 Bohr^{-2} . As a result, the repulsive force between *OOH and N_4 are enhanced, which is in responsible to the weakened Co-OOH bonding in electron-rich Co site of CoPc-S-COF.

2. Supplementary Figures and Tables.

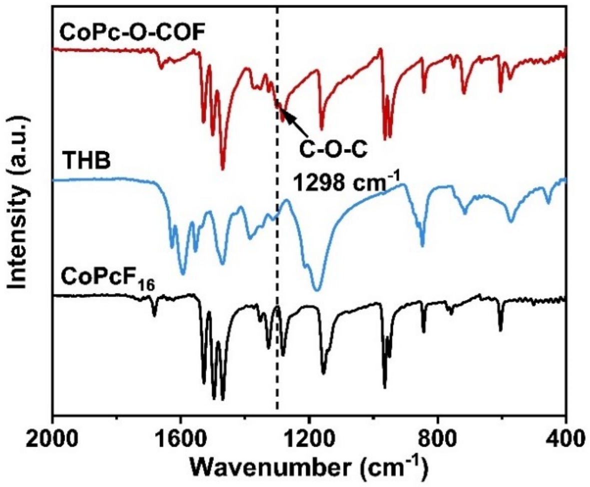

Supplementary Fig. 1. FT-IR spectra of $CoPcF_{16}$, THB, and CoPc-O-COF.

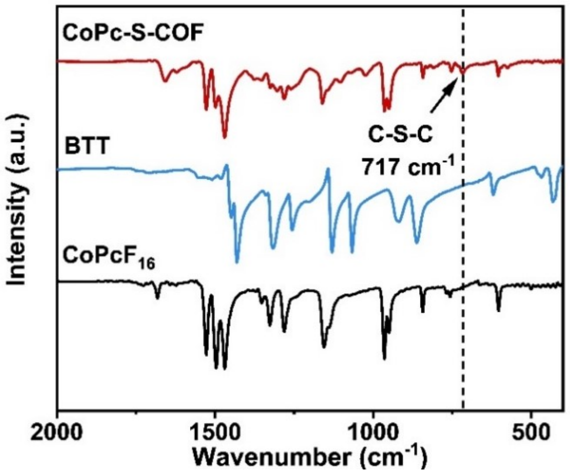

Supplementary Fig. 2. FT-IR spectra of $CoPcF_{16}$, BTT, and CoPc-S-COF.

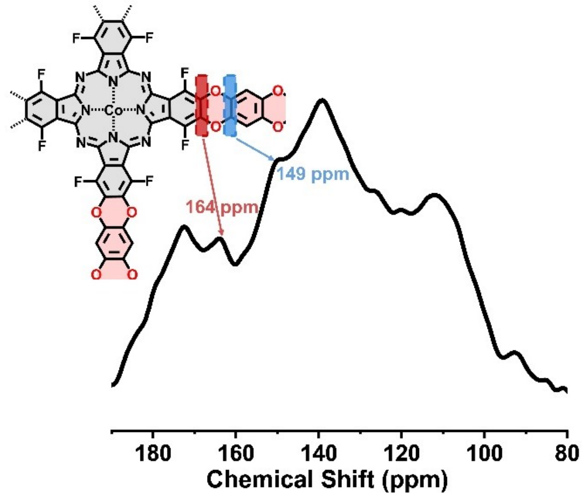

Supplementary Fig. 3. The solid-state ^{13}C CP/MAS NMR spectrum of CoPc-O-COF.

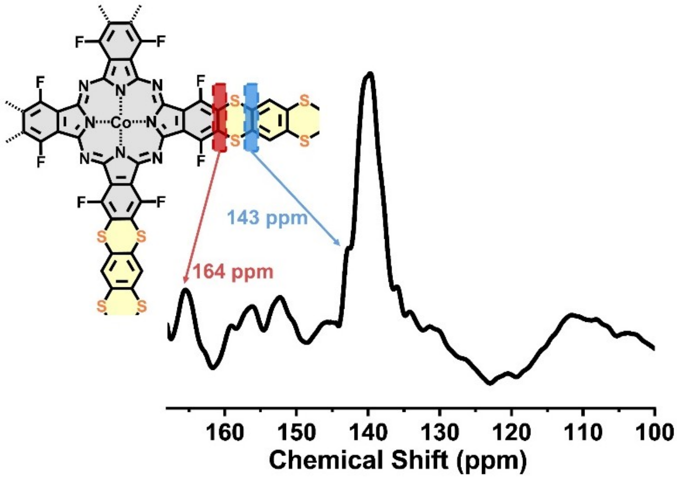

Supplementary Fig. 4. The solid-state ^{13}C CP/MAS NMR spectrum of CoPc-S-COF.

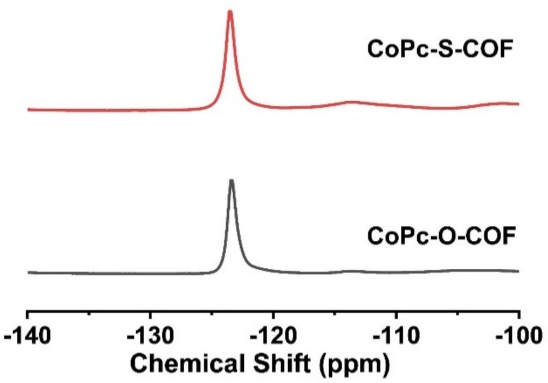

Supplementary Fig. 5. The solid-state ^{19}F CP/MAS NMR spectra of CoPc-O-COF and CoPc-S-COF.

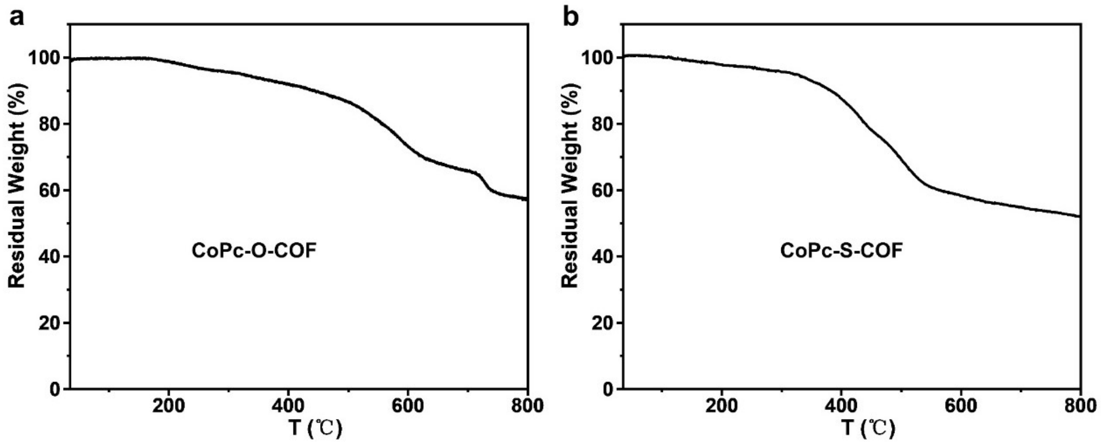

Supplementary Fig. 6. TGA of (a) CoPc-O-COF and (b) CoPc-S-COF.

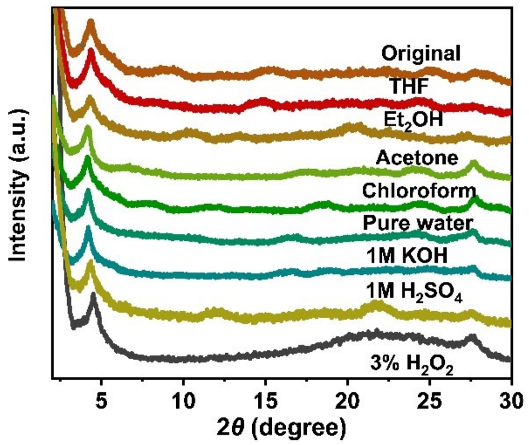

Supplementary Fig. 7. PXRD patterns of CoPc-S-COF after immersion in different solutions.

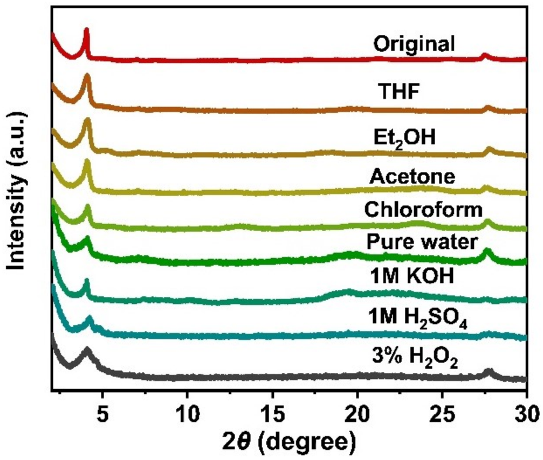

Supplementary Fig. 8. PXRD patterns of CoPc-O-COF after immersion in different solutions.

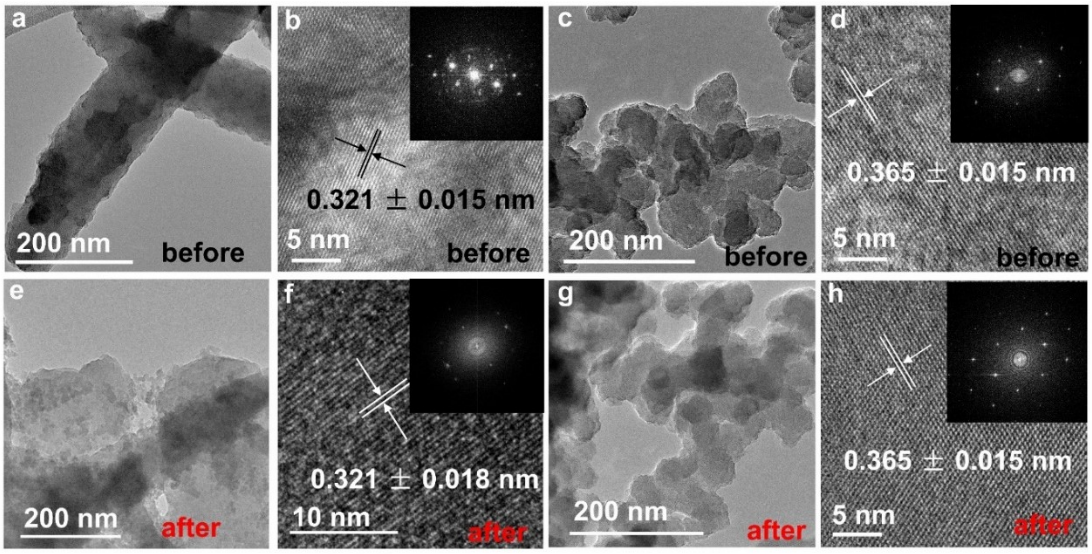

Supplementary Fig. 9. TEM and HR-TEM images of (a, b, e, f) CoPc-O-COF and (c, d, g, h) CoPc-S-COF before and after soaking into 3% H_2O_2 solution for three days.

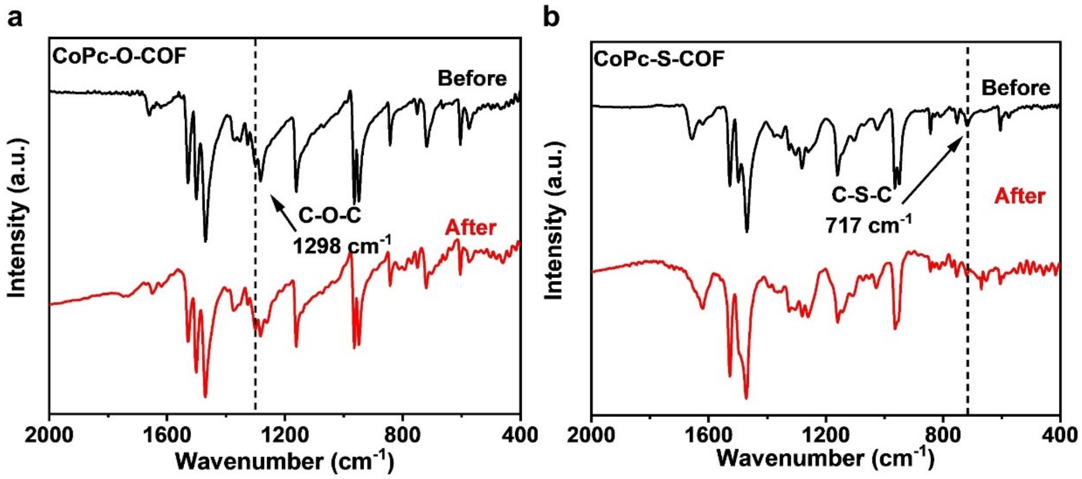

Supplementary Fig. 10. FT-IR spectra of (a) CoPc-O-COF and (b) CoPc-S-COF before and after soaking into 3% H_2O_2 solution for three days.

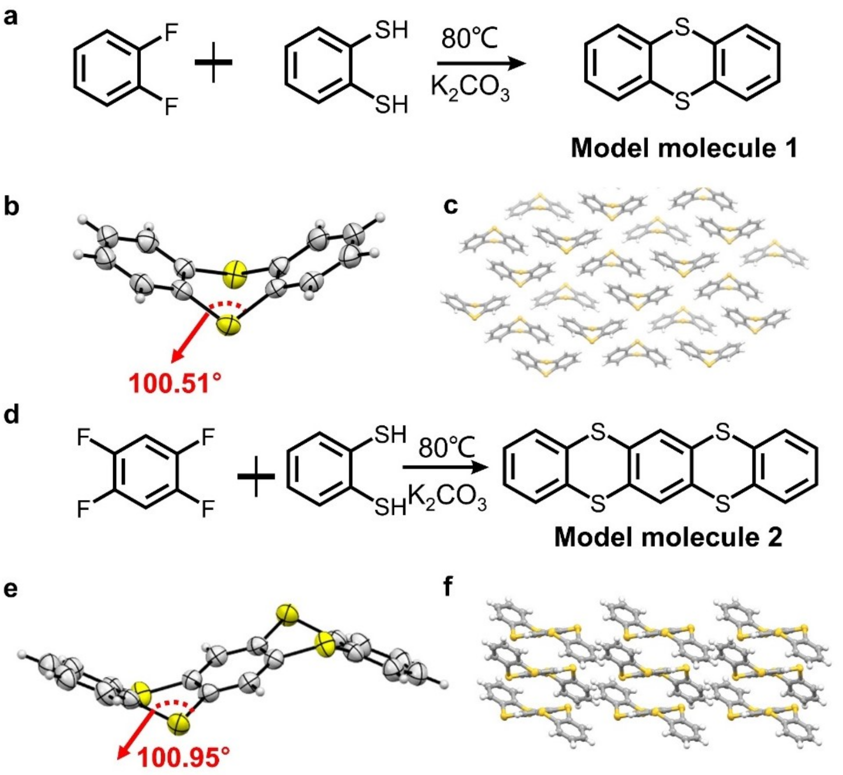

Supplementary Fig. 11 (a, d) Schematic synthesis of the model molecules. (b, e) Molecular and (c, f) packing structure of the model molecules in their single crystals. C, H, and S are displayed in grey, white, and yellow, respectively.

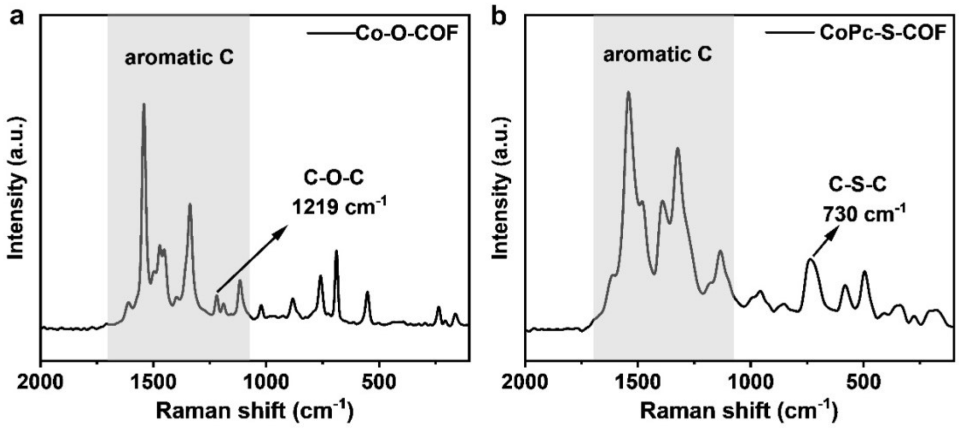

Supplementary Fig. 12. Raman spectra of (a) CoPc-O-COF and (b) CoPc-S-COF.

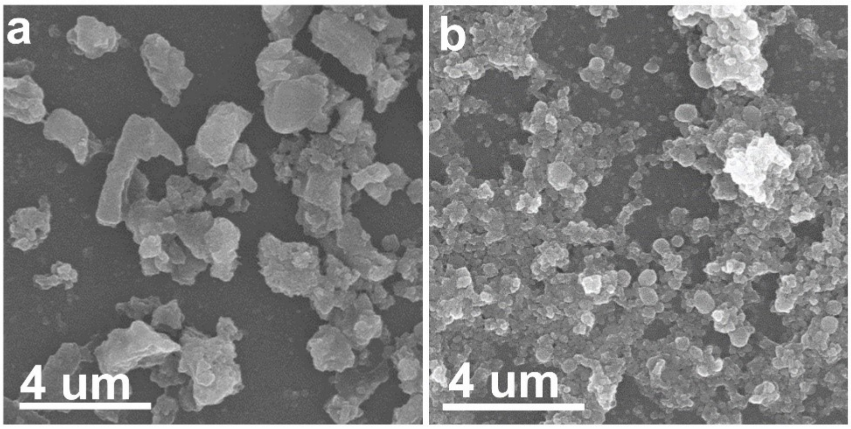

Supplementary Fig. 13. SEM images of (a) CoPc-O-COF and (b) CoPc-S-COF.

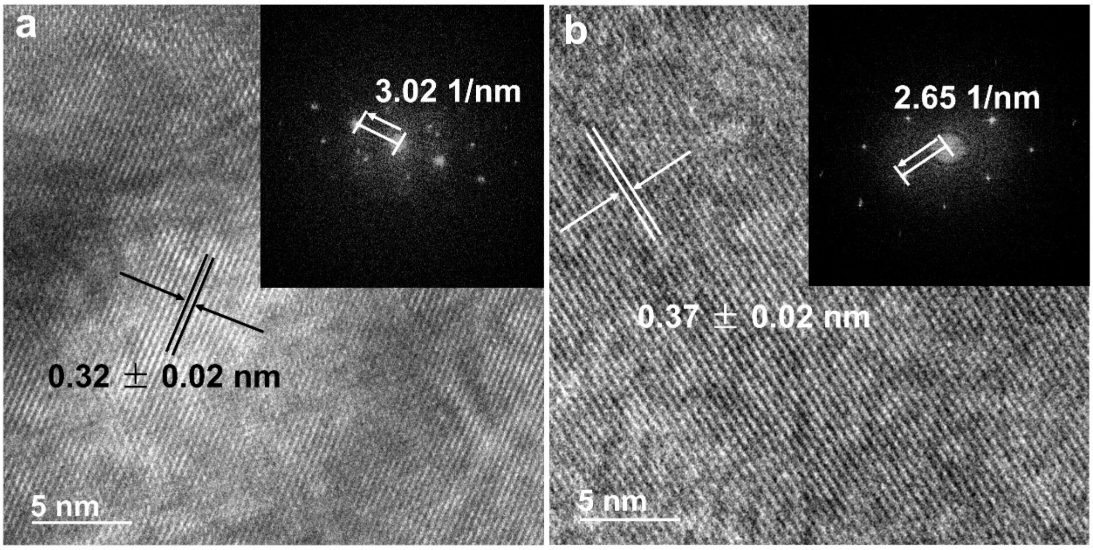

Supplementary Fig. 14. HRTEM images of (a) CoPc-O-COF and (b) CoPc-S-COF. Inset: the related FFT analysis.

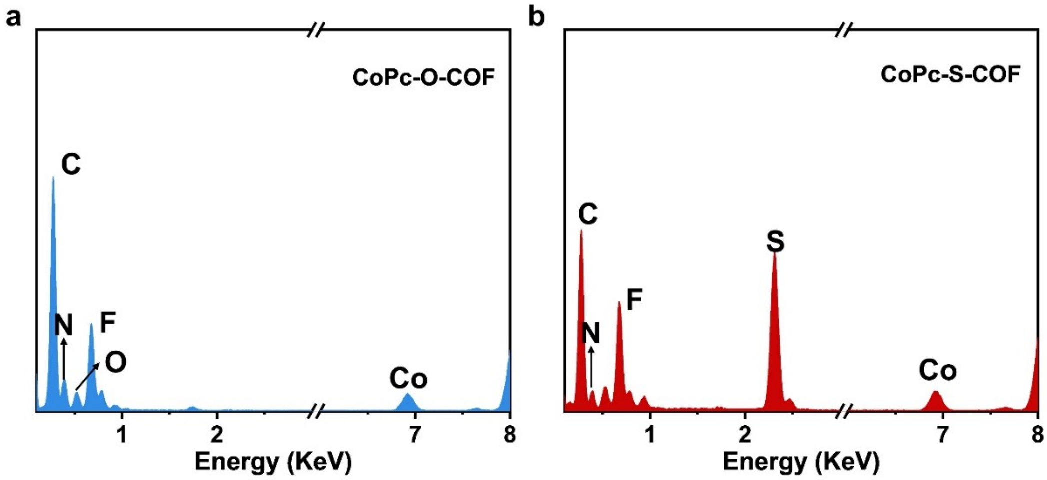

Supplementary Fig. 15. EDX data of (a) CoPc-O-COF and (b) CoPc-S-COF.

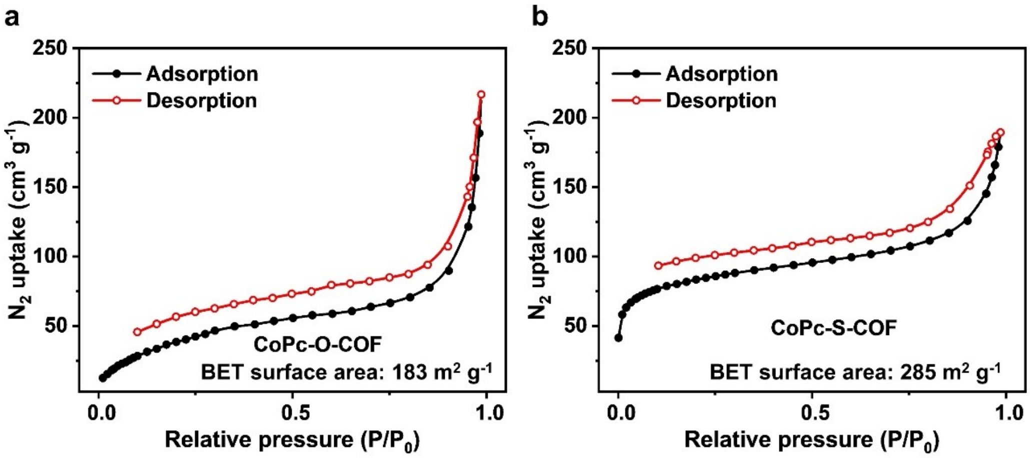

Supplementary Fig. 16. N_2 adsorption–desorption isotherms of (a) CoPc-O-COF and (b) CoPc-S-COF.

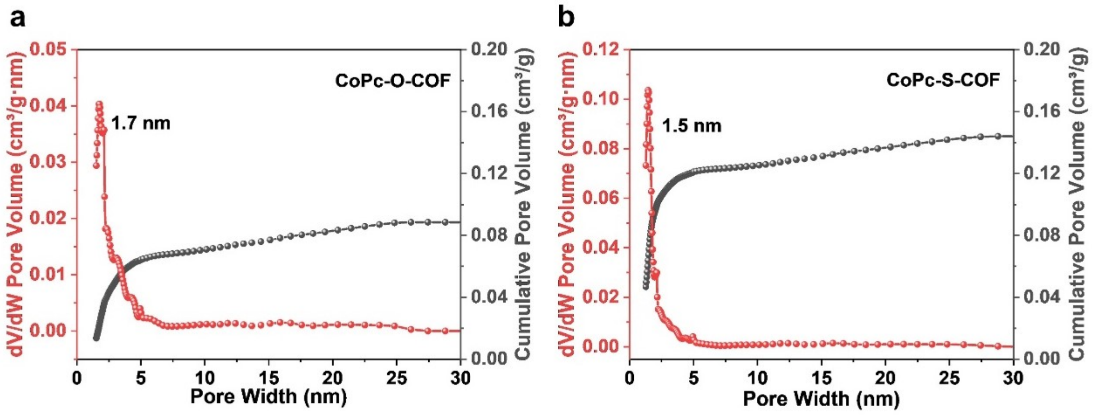

Supplementary Fig. 17. Pore volume and pore size distribution of (a) CoPc-O-COF and (b) CoPc-S-COF.

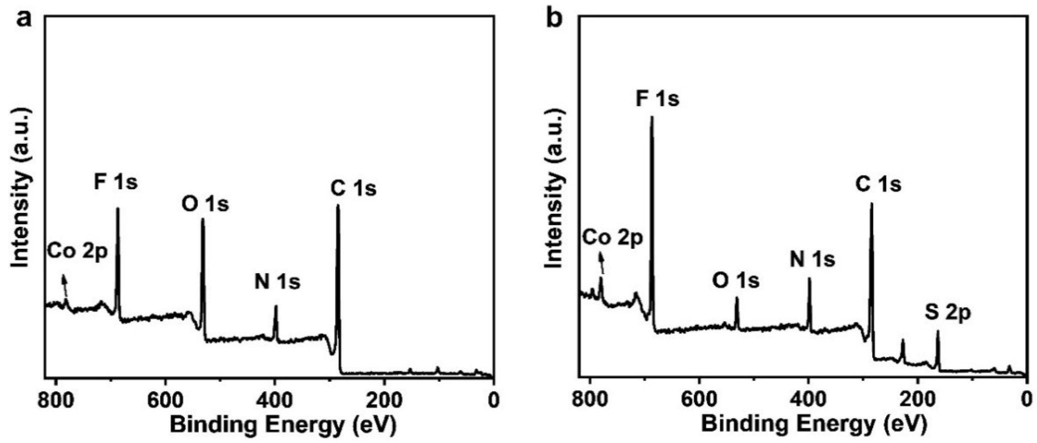

Supplementary Fig. 18. XPS survey spectra of (a) CoPc-O-COF and (b) CoPc-S-COF.

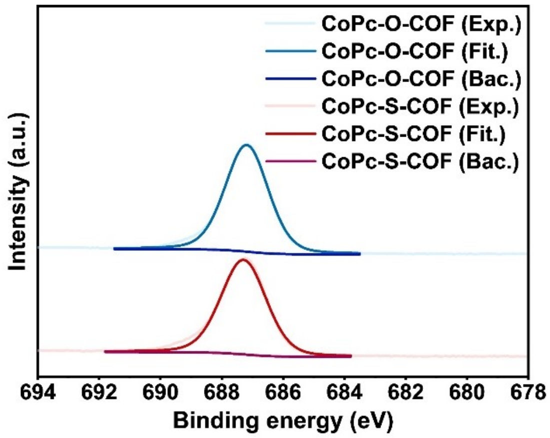

Supplementary Fig. 19. F 1*s* XPS spectra of CoPc-O-COF and CoPc-S-COF.

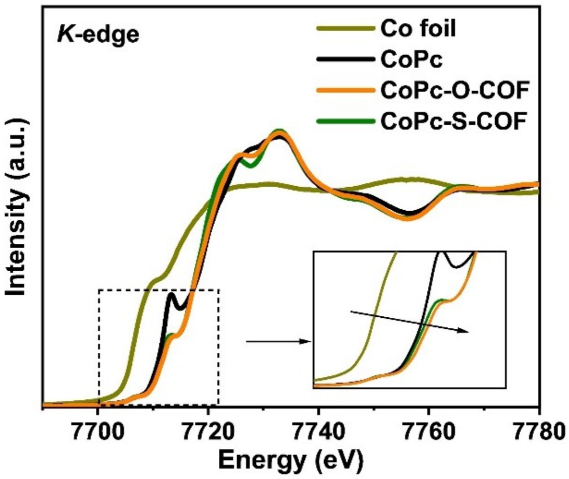

Supplementary Fig. 20. Co *K*-edge XANES spectra of CoPc, CoPc-O-COF, CoPc-S-COF, and Co foil.

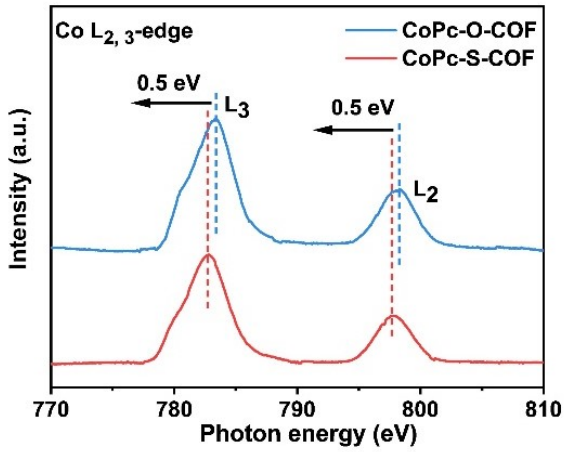

Supplementary Fig. 21. Co $L_{2,\ 3}$-edge XANES spectra of CoPc-O-COF and CoPc-S-COF.

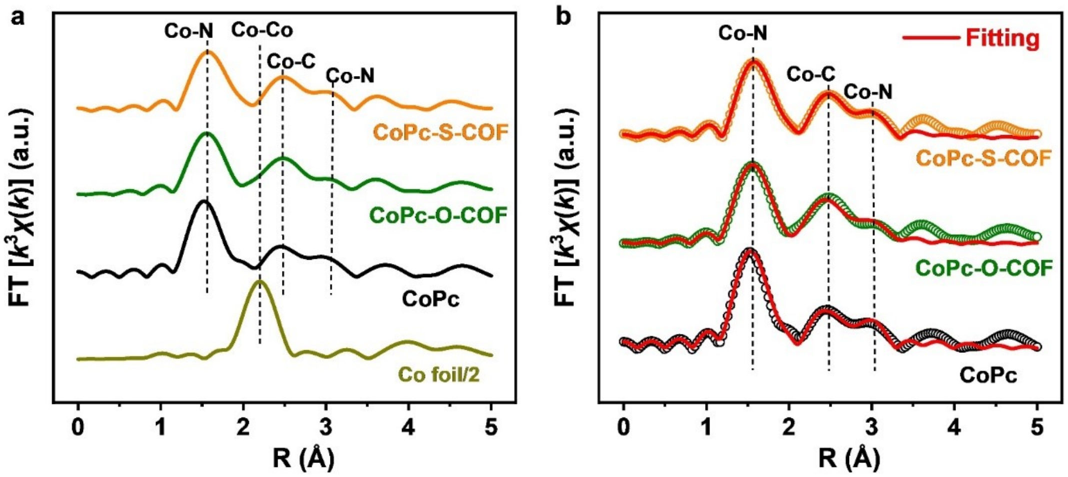

Supplementary Fig. 22. (a) Fourier transform of EXAFS spectra of CoPc, Co foil, CoPc-O-COF, and CoPc-S-COF in R space. (b) The EXAFS fitting results of CoPc-O-COF, CoPc-S-COF and CoPc in R space.

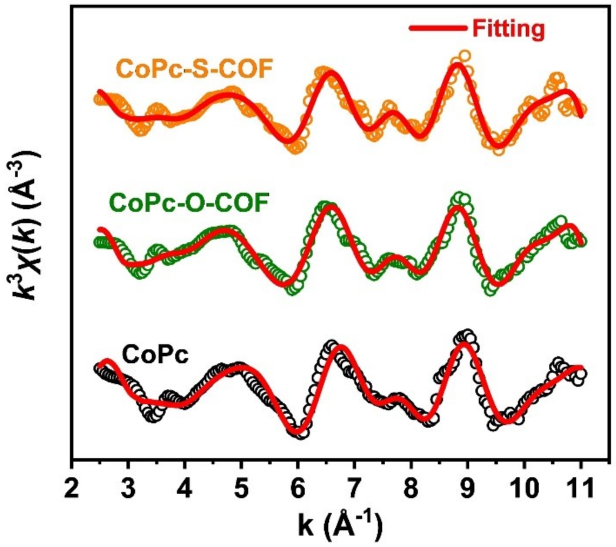

Supplementary Fig. 23. The Co k^3-weighted *K*-space spectra of CoPc-O-COF, CoPc-S-COF and CoPc.

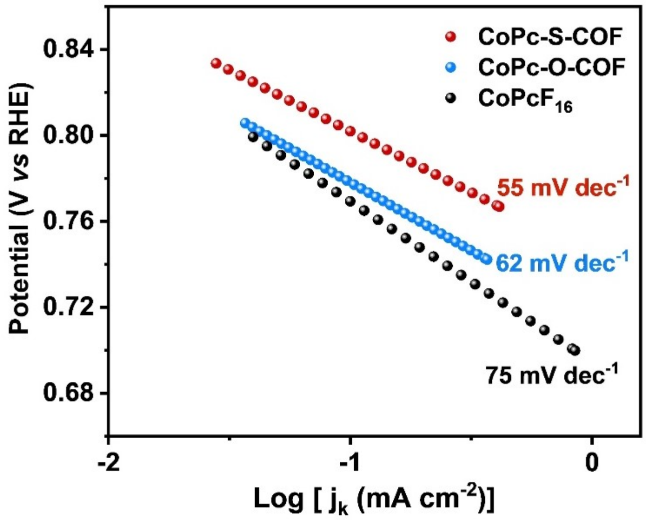

Supplementary Fig. 24. Tafel plots of CoPc-O-COF, CoPc-S-COF and $CoPcF_{16}$.

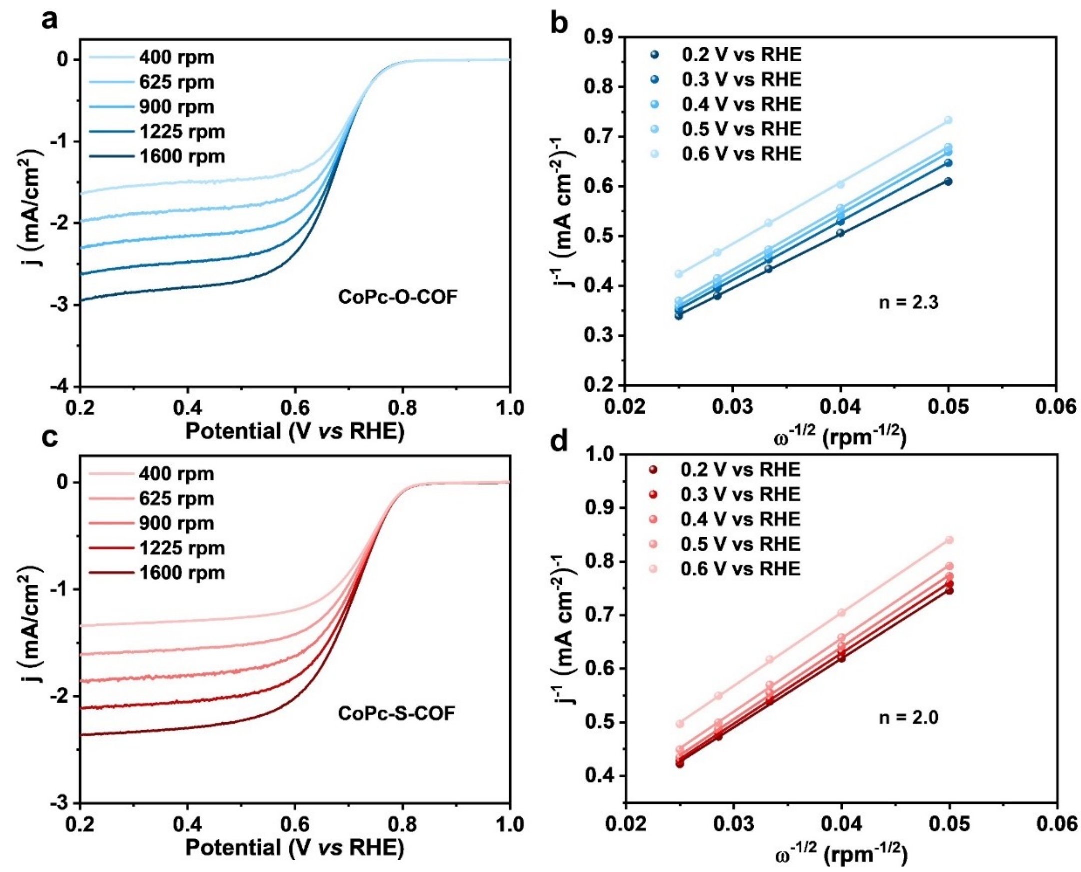

Supplementary Fig. 25. ORR polarization curves of (a) CoPc-O-COF and (c) CoPc-S-COF at different rotate rates. K–L plots of (b) CoPc-O-COF and (d) CoPc-S-COF.

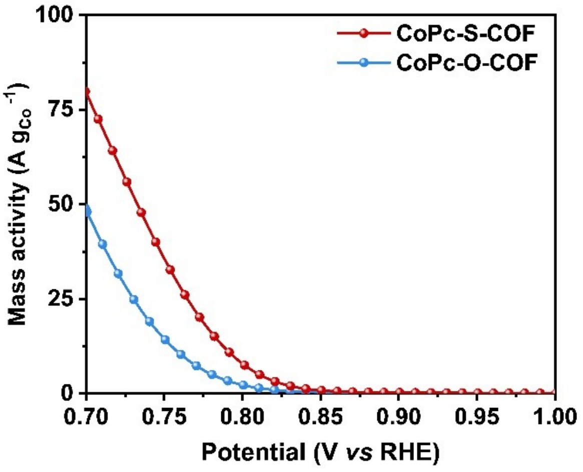

Supplementary Fig. 26. Mass activity of CoPc-O-COF and CoPc-S-COF.

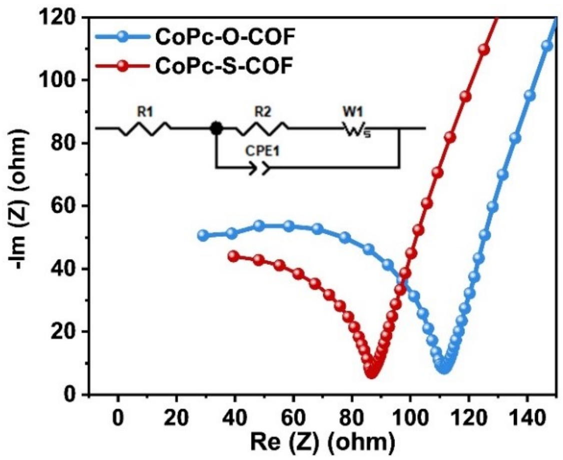

Supplementary Fig. 27. EIS plots of CoPc-O-COF and CoPc-S-COF. Inset: equivalent circuit diagram for fitting results. (R_s: solution resistance. R_{ct}: charge transfer resistance. W_1: spreading resistance. CPE1: constant phase angle element.)

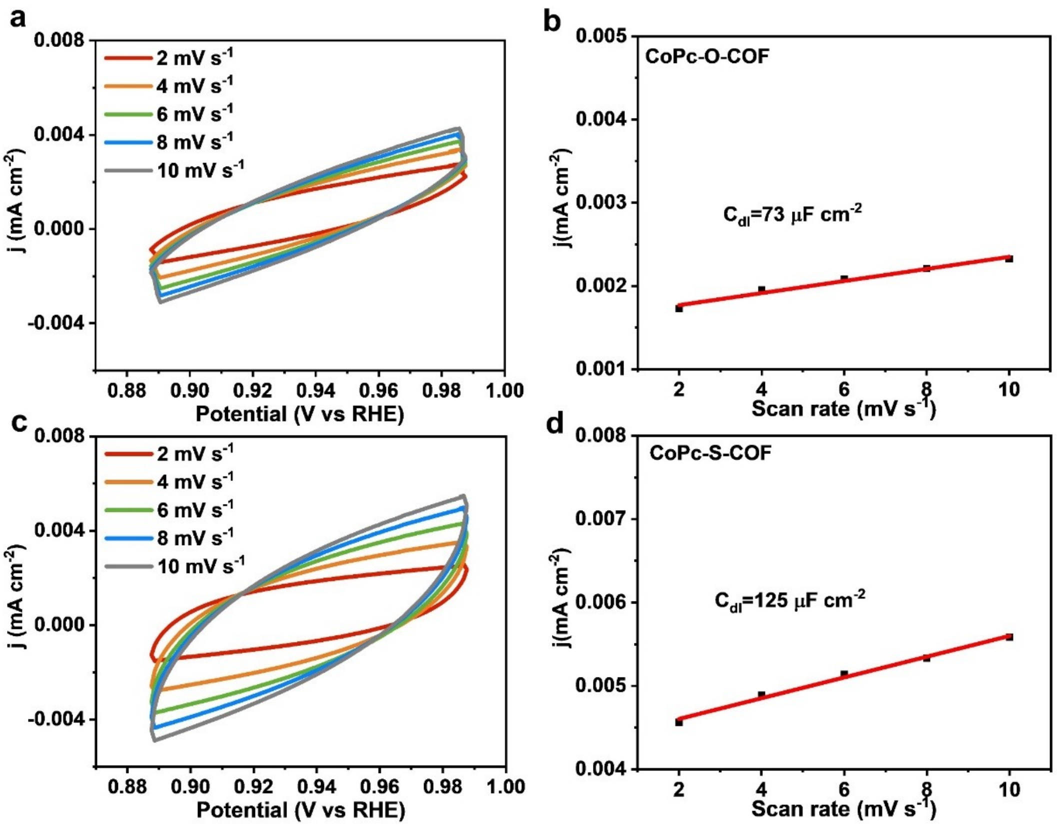

Supplementary Fig. 28. CV curves at different scanning rates of (a) CoPc-O-COF and (c) CoPc-S-COF. The calculated C_{dl} for (b) CoPc-O-COF and (d) CoPc-S-COF.

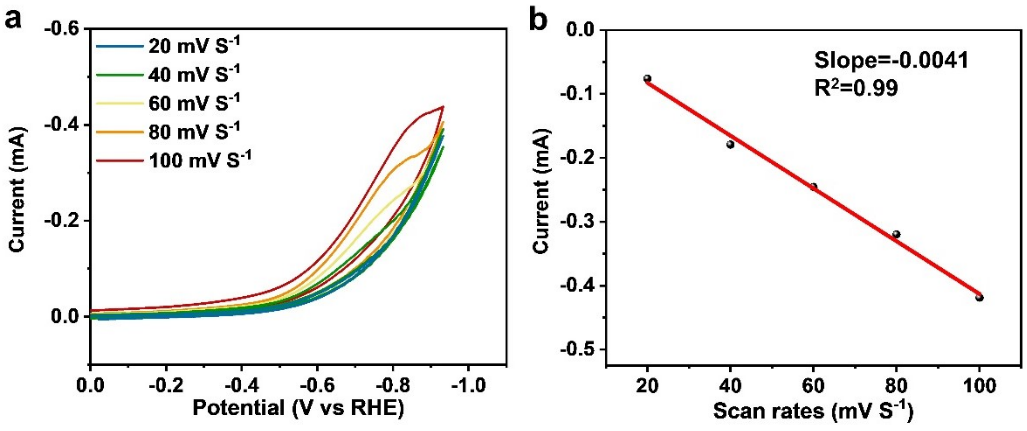

Supplementary Fig. 29. (a) Scan rate dependence of CV response of CoPc-S-COF. (b) Peak current of CVs for CoPc-S-COF as a function of scan rate.

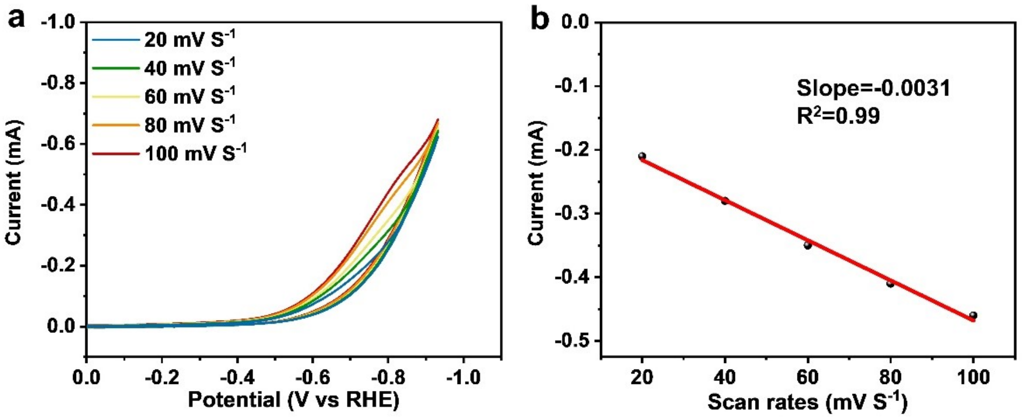

Supplementary Fig. 30. (a) Scan rate dependence of CV response of CoPc-O-COF. (b) Peak current of CVs for CoPc-O-COF as a function of scan rate.

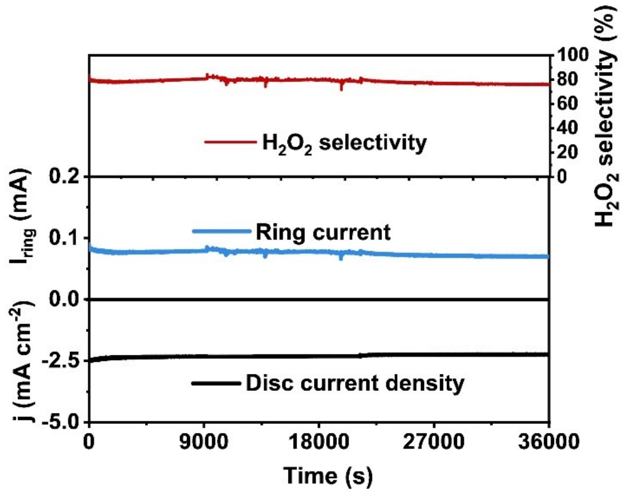

Supplementary Fig. 31. Chronoamperometry measurement of the CoPc-O-COF for 36000 s at 0.52 V vs RHE.

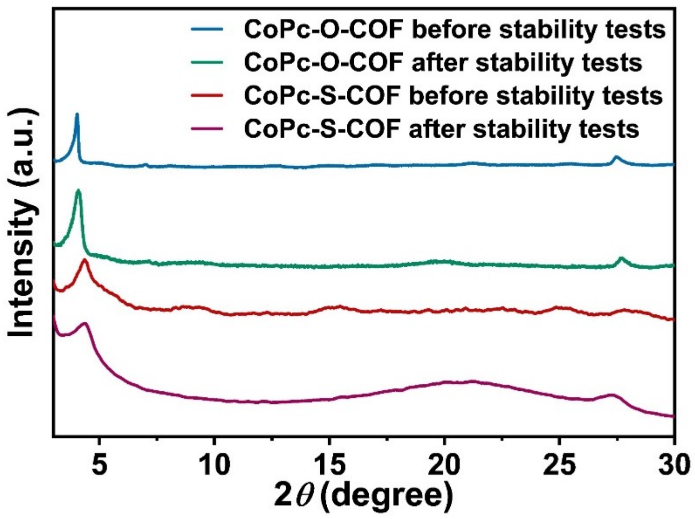

Supplementary Fig. 32. PXRD patterns of CoPc-O-COF and CoPc-S-COF before and after stability tests.

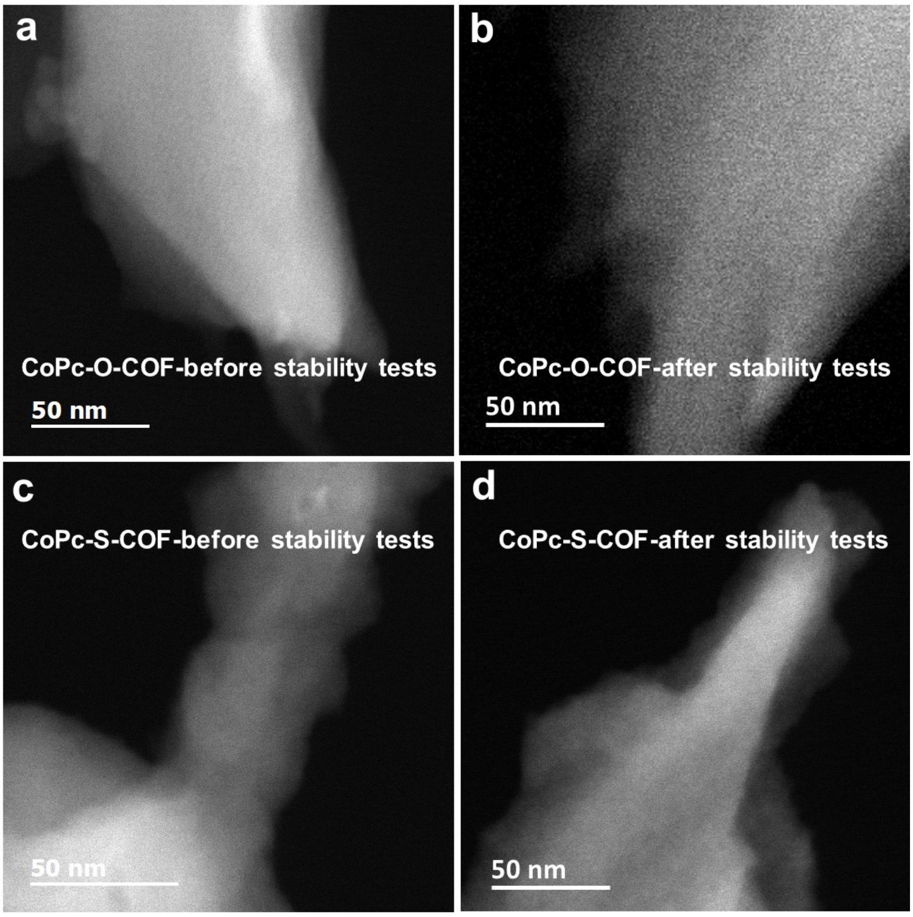

Supplementary Fig. 33. STEM images of (a, b) CoPc-O-COF and (c, d) CoPc-S-COF before and after stability tests.

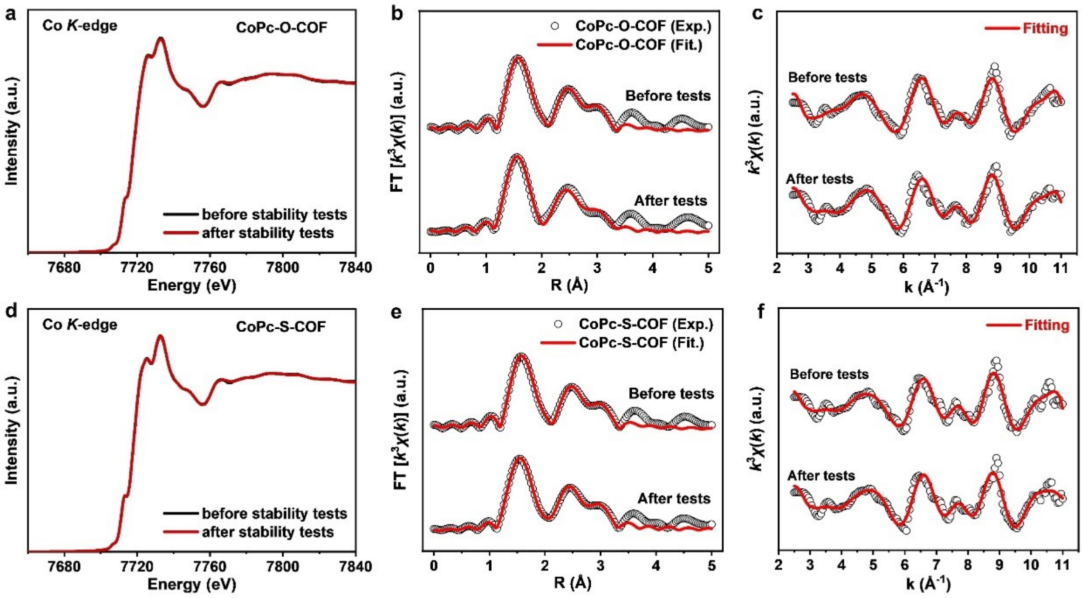

Supplementary Fig. 34. (a, d) The Co K-edge XANES spectra of CoPc-O-COF and CoPc-S-COF before and after stability tests. (b, e) The EXAFS fitting results of CoPc-O-COF and CoPc-S-COF before and after stability tests. (c, f) The Co k^3-weighted *K*-space spectra of CoPc-O-COF and CoPc-S-COF before and after stability tests.

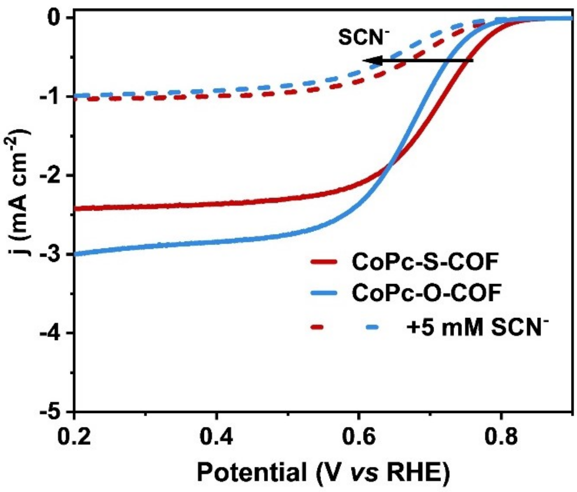

Supplementary Fig. 35. ORR polarization curves in 0.1 M KOH before and after the addition of 5 mM SCN^-.

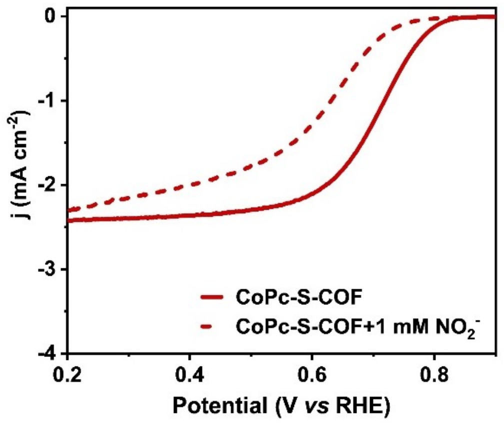

Supplementary Fig. 36. ORR polarization curves in 0.1 M KOH before and after the addition of 1 mM nitrite into the electrolyte.

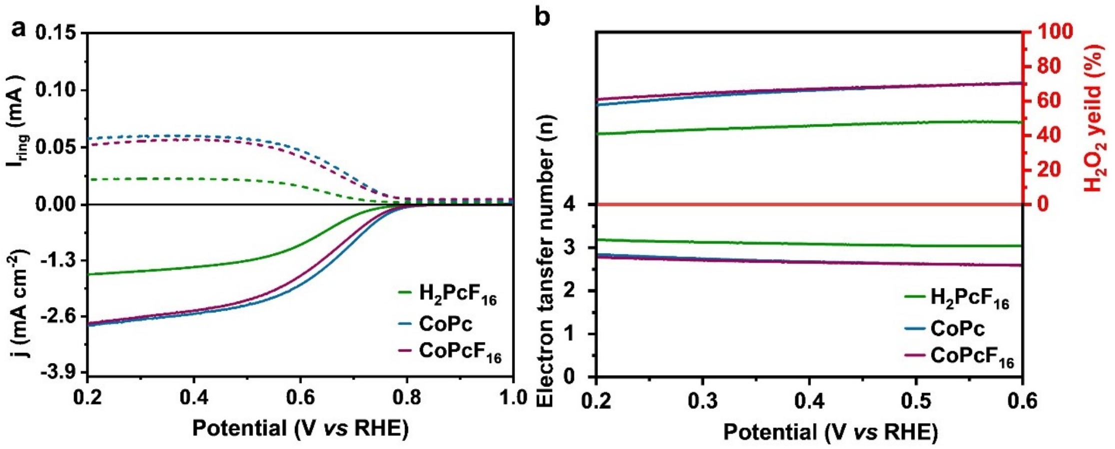

Supplementary Fig. 37. (a) The LSVs of CoPc, $CoPcF_{16}$, and H_2PcF_{16} at 1600 rpm in O_2-saturated 0.1 M KOH. (b) H_2O_2 selectivity and electron transfer number n of CoPc, $CoPcF_{16}$, and H_2PcF_{16}.

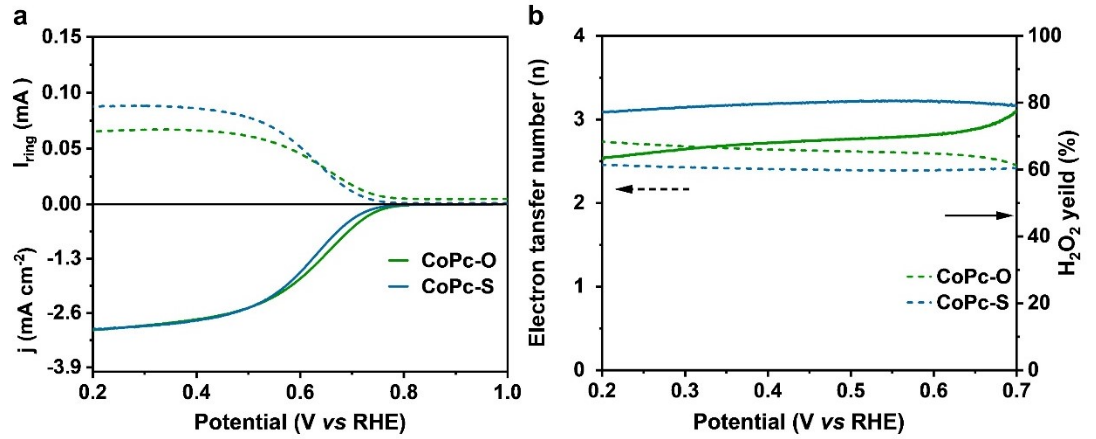

Supplementary Fig. 38. (a) The LSVs of CoPc-O and CoPc-S at 1600 rpm in O_2-saturated 0.1 M KOH. (b) H_2O_2 selectivity and electron transfer number n of CoPc-O and CoPc-S.

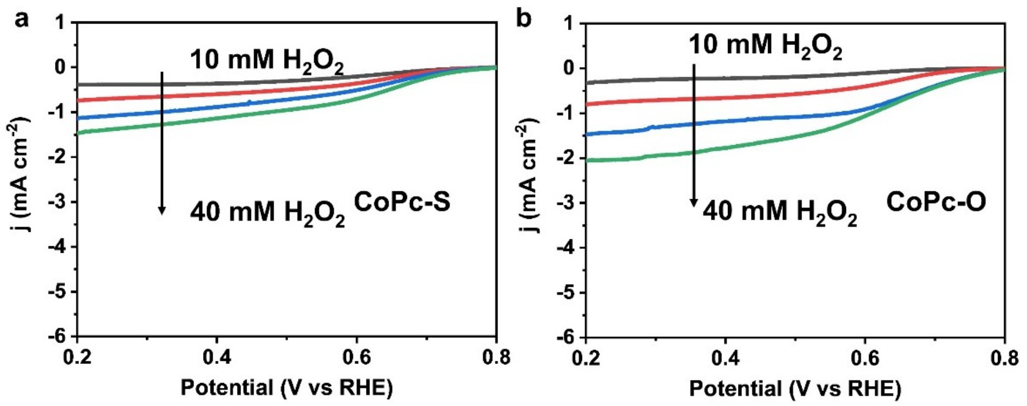

Supplementary Fig. 39. H_2O_2RR polarization curves of (a) CoPc-S and (b) CoPc-O in Ar-saturated 0.1 M KOH containing different concentrations of H_2O_2.

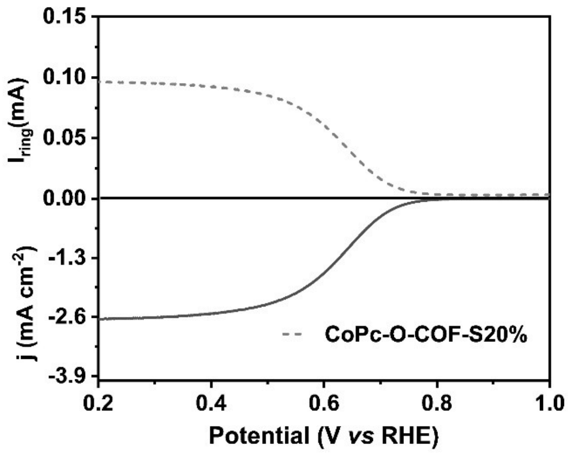

Supplementary Fig. 40. The LSVs of CoPc-O-COF-S 20% at 1600 rpm in O_2-saturated 0.1 M KOH.

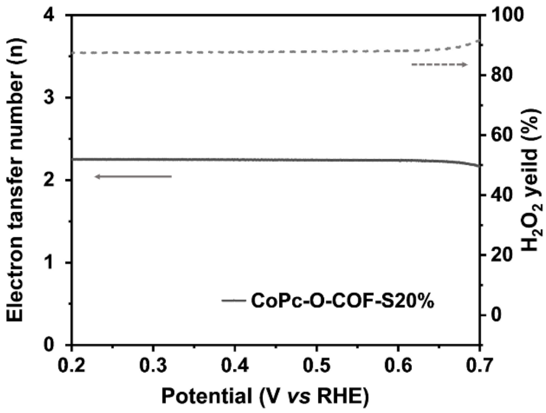

Supplementary Fig. 41. H_2O_2 selectivity and electron transfer number n of CoPc-O-COF-S20%.

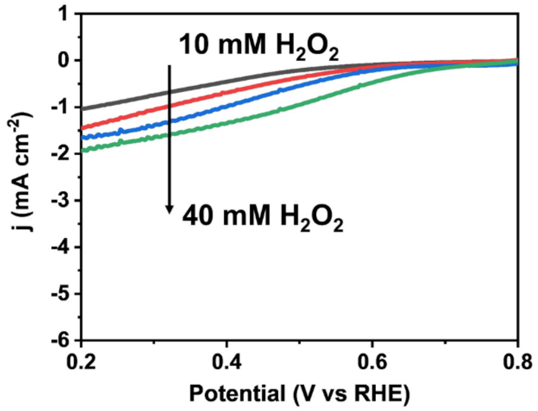

Supplementary Fig. 42. (a) H_2O_2RR polarization curves of CoPc-O-COF-S20% in Ar-saturated 0.1 M KOH containing different concentrations of H_2O_2.

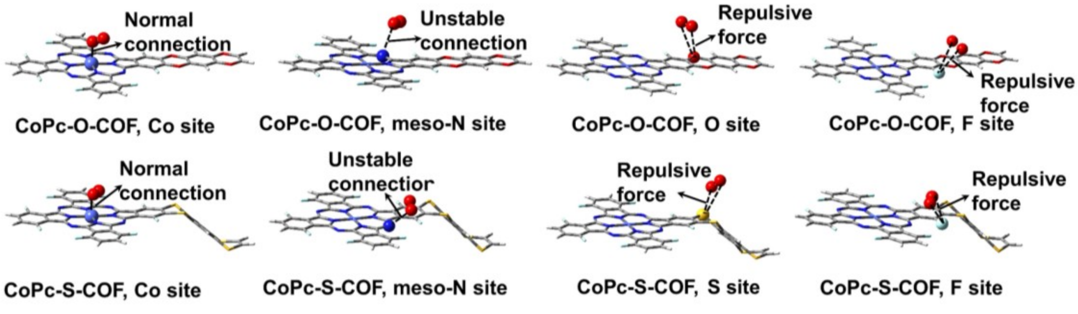

Supplementary Fig. 43. The schematic diagrams of adsorbed O_2 molecule on various sites of the two COFs including Co, N, O, S, and F atoms.

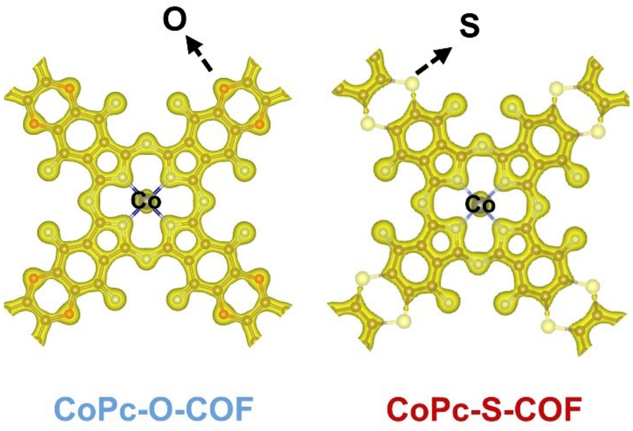

Supplementary Fig. 44. Electron density distribution on the simulated periodic fragment of CoPc-O-COF and CoPc-S-COF (lsosurface level: 0.20).

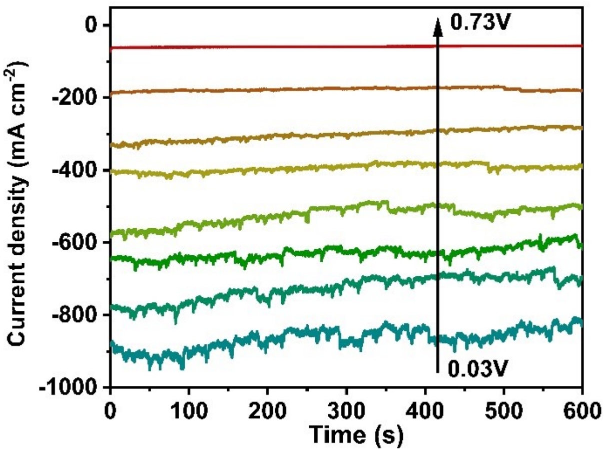

Supplementary Fig. 45. The chronoamperometry measurements at varied applied voltages of CoPc-O-COF.

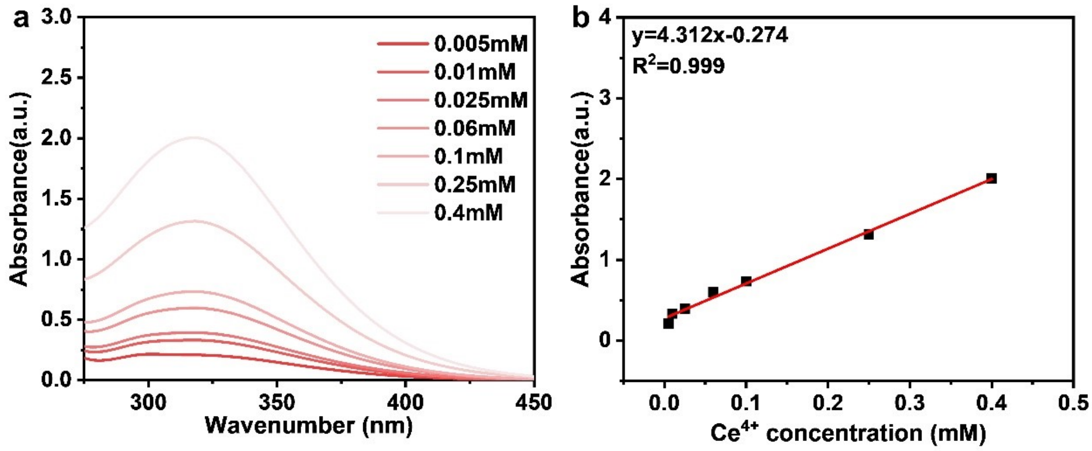

Supplementary Fig. 46. (a) The UV-Vis spectra of Ce^{4+} solutions with different concentrations. (b) The linear calibration relationship between Ce^{4+} concentration and corresponding absorbance peak at 318 nm.

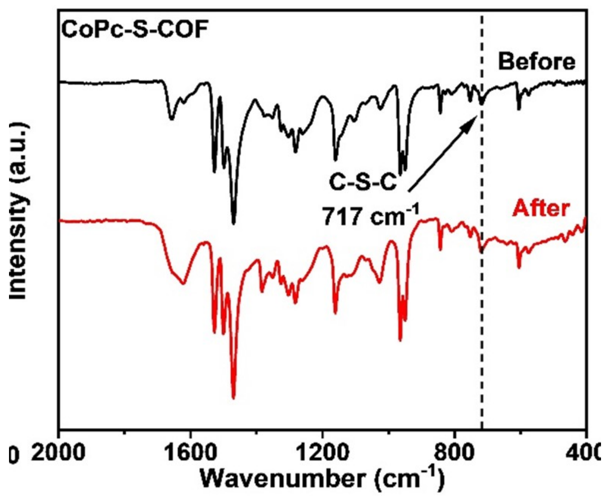

Supplementary Fig. 47. FT-IR spectra of CoPc-S-COF before and after stability tests.

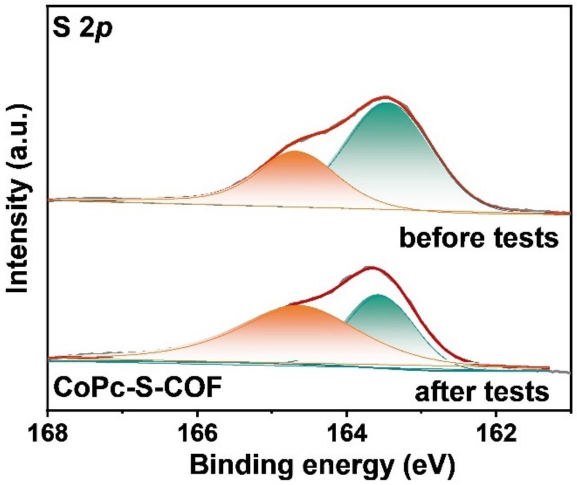

Supplementary Fig. 48. High-resolution S 2*p* XPS spectra of CoPc-S-COF before and after stability tests.

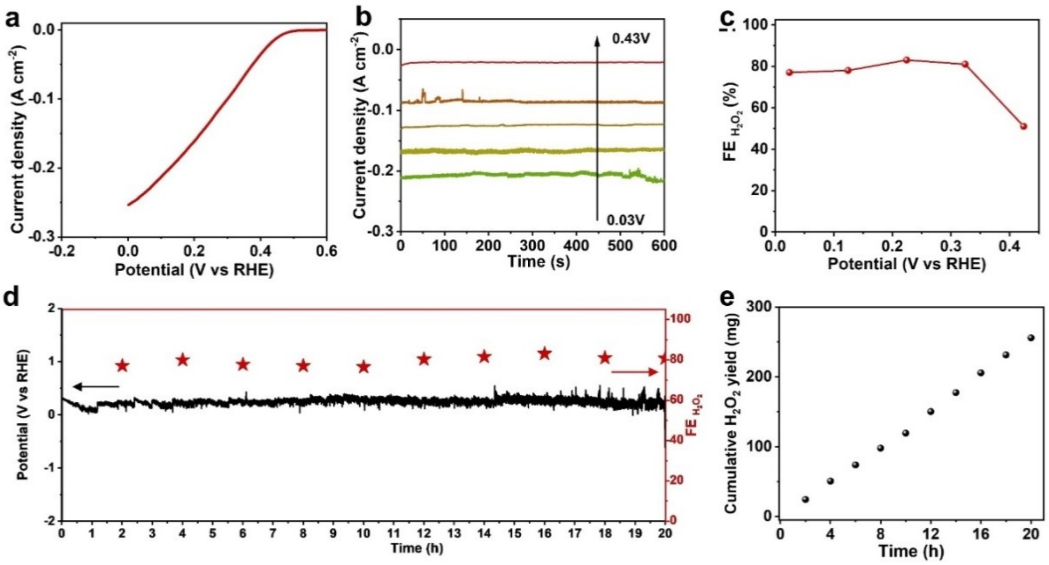

Supplementary Fig. 49. **H_2O_2 electroproduction in the flow cell with 1 M Na_2SO_4 electrolyte (pH = 7).** (a) LSV of CoPc-S-COF in flow cell. (b) The chronoamperometry measurements at varied applied voltages of CoPc-S-COF. (c) FE_{H2O2} of CoPc-S-COF at varied applied voltages. (e) H_2O_2 yields of CoPc-S-COF. (d) Chronopotentiometry curve at a current density of 100 mA cm^{-2} and the corresponding FE_{H2O2} in the flow cell for CoPc-S-COF.

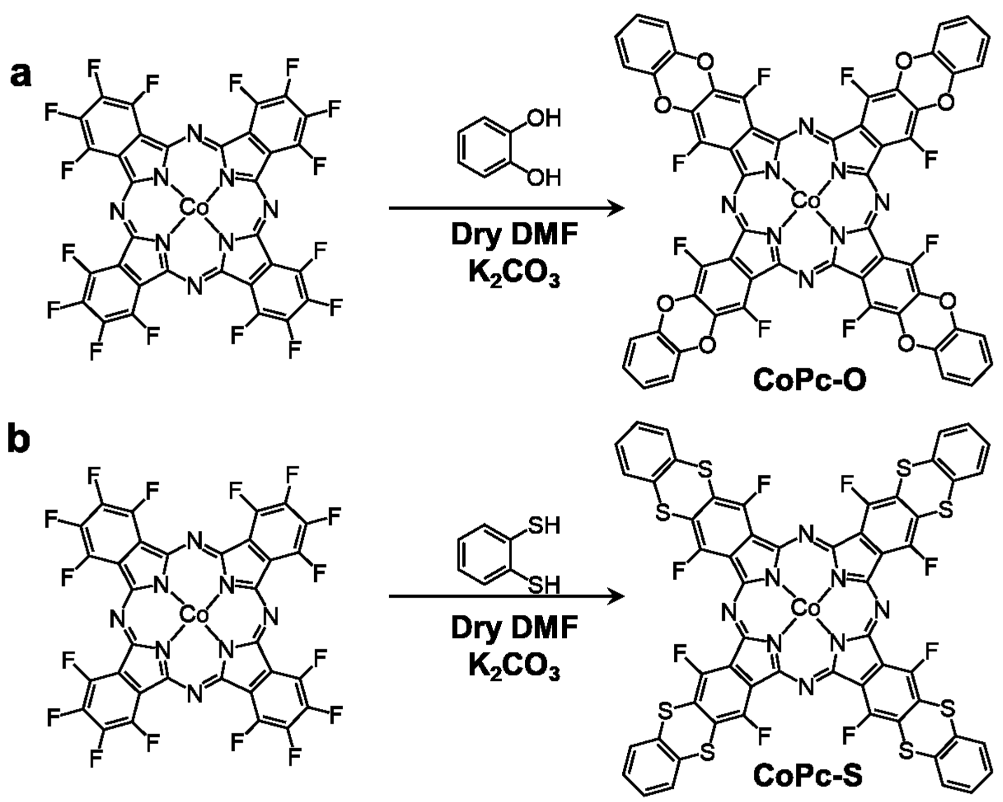

Supplementary Fig. 50. Synthesis of (a) CoPc-O and (b) CoPc-S.

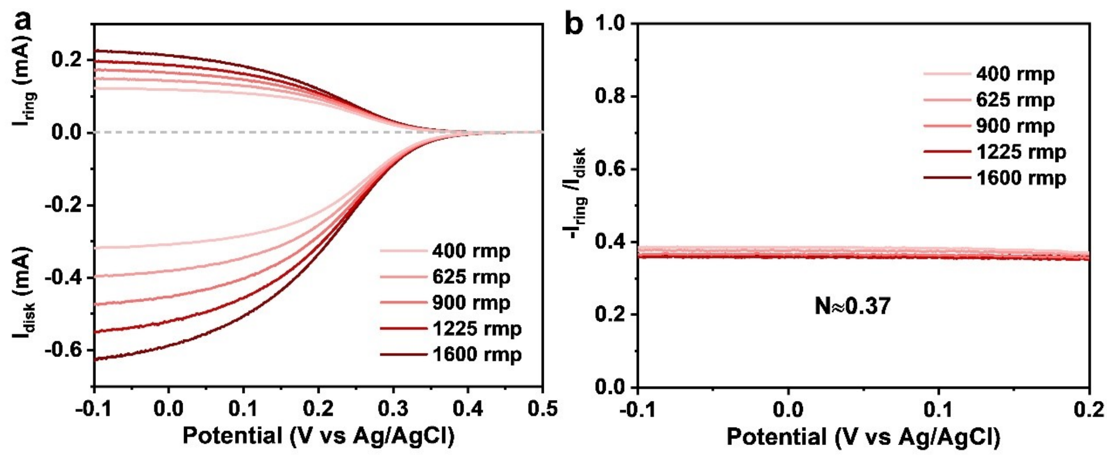

Supplementary Fig. 51. (a) The LSV curves in 1 M KCl + 10 mM $K_3[Fe(CN)_6]$ at different rotate speeds. (b) Determination of the collection efficiency (N) for RRDE.

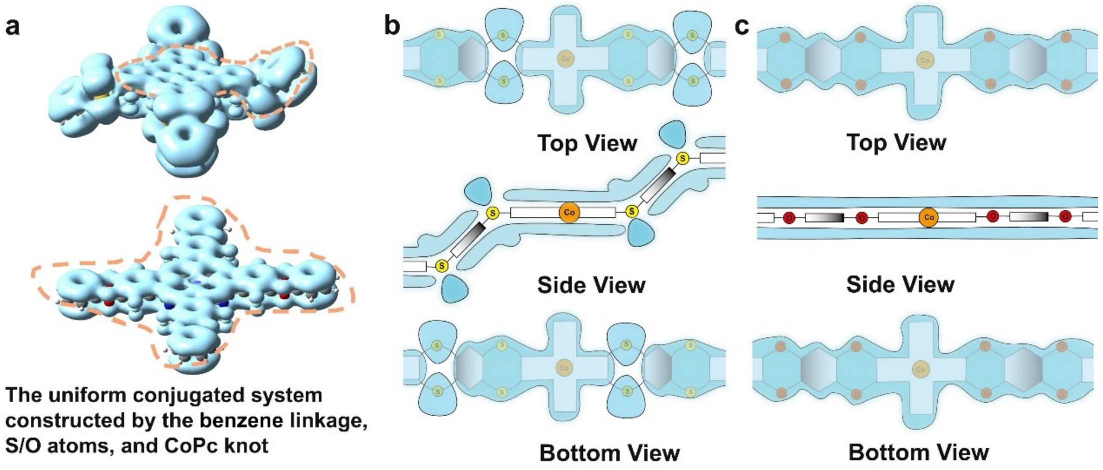

Supplementary Fig. 52. (a) The visualized scheme of the uniform conjugated system of CoPc-S-COF (top) and CoPc-O-COF (bottom). The schematic diagram of the uniform conjugated system in top/side/bottom view for (b) CoPc-S-COF and (c) CoPc-O-COF.

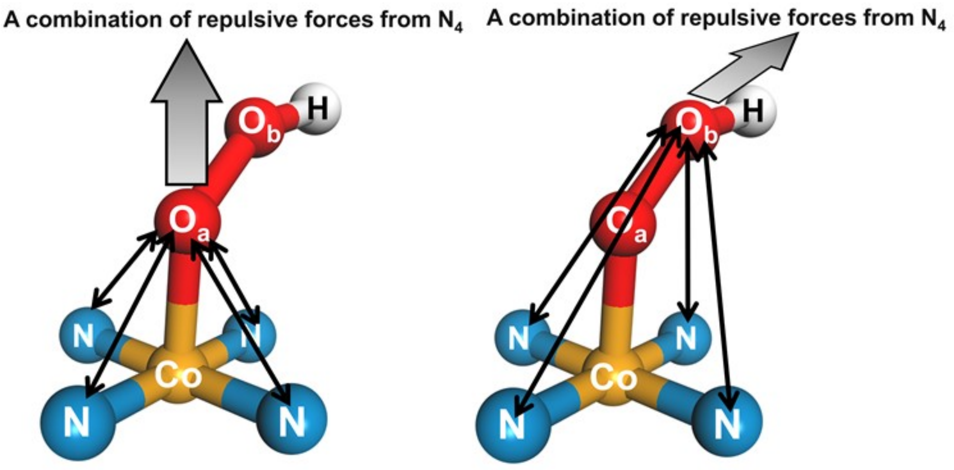

Supplementary Fig. 53. The schematic drawing of enhanced repulsive force between *OOH and N_4.

Supplementary Table 1. Single crystal X-ray diffraction experimental details.

	**Model molecule 1**	**Model molecule 2**
CCDC number	2302889	2305173
Empirical formula	$C_{12}H_8S_2$	$C_{18}H_{10}S_4$
Formula weight	216.30	354.50
Temperature [K]	293(2)	293(2)
Crystal system	monoclinic	monoclinic
Space group (number)	$P2_1/c$ (14)	*C 2/c*
a [Å]	11.9449(8)	21.076(2)
b [Å]	6.1481(4)	10.0464(8)
c [Å]	14.4785(10)	7.4290(7)
α [°]	90	90
β [°]	109.998(8)	96.849(9)
γ [°]	90	90
Volume [Å^3]	999.17(13)	1561.8(2)
Z	4	4
ρ_{calc} [gcm^{-3}]	1.438	1.508
μ [mm^{-1}]	0.483	0.600
F(000)	448	728
Radiation	Mo K_α (λ=0.71073 Å)	Mo K_α (λ=0.71073 Å)
2Θ range [°]	5.85 to 58.23 (0.73 Å)	4.50 to 58.43 (0.73 Å)
Index ranges	$-11 \leq h \leq 16$<br>$-8 \leq k \leq 8$<br>$-18 \leq l \leq 19$	$-28 \leq h \leq 25$<br>$-12 \leq k \leq 13$<br>$-10 \leq l \leq 10$
Reflections collected	4567	8492
Independent reflections	2249<br>R_{int} = 0.0213<br>R_{sigma} = 0.0355	1903<br>R_{int} = 0.1091<br>R_{sigma} = 0.0752
Completeness	98.7 %	99.9%
Data / Restraints / Parameters	2249/0/127	2249/0/100
Goodness-of-fit on F^2	1.054	1.028
Final *R* indexes [$I \geq 2\sigma(I)$]	R_1 = 0.0354<br>wR_2 = 0.0822	R_1 = 0.0703<br>wR_2 = 0.1719
Final *R* indexes [all data]	R_1 = 0.0482<br>wR_2 = 0.0873	R_1 = 0.0937<br>wR_2 = 0.1890
Largest peak/hole [eÅ^{-3}]	0.20/-0.27	0.99/-0.31

Supplementary Table 2. The atomic ratios form EDX mapping analysis of CoPc-O-COF and CoPc-S-COF.

Sample		C (at%)	N (at%)	O (at%)	S (at%)	F (at%)	Co (at%)
**CoPc-O-COF**	Calculated	63.7	11.6	11.6	-	11.6	1.4
	Tested	63.6	10.3	13.5	-	12.2	1.3
**CoPc-S-COF**	Calculated	63.7	11.6	-	11.6	11.6	1.4
	Tested	62.5	10.5		11.9	13.5	1.5

Supplementary Table 3. The atom radios of CoPc-O-COF and CoPc-S-COF before and after stability tests by the measurements of XPS.

Sample		C (at%)	N (at%)	O (at%)	S (at%)	F (at%)	Co (at%)
**CoPc-O-COF**	Calculated	63.7	11.6	11.6	-	11.6	1.4
	Before	59.9	11.4	12.8	-	12.5	1.2
	After	60.2	11.8	12.4	-	11.7	1.6
**CoPc-S-COF**	Calculated	63.7	11.6	-	11.6	11.6	1.4
	Before	60.4	10.5	-	10.6	14.2	1.2
	After	61.0	10.6	-	12.2	11.2	1.0

Supplementary Table 4. The fitting parameters of the Fourier transform EXAFS spectra.

Sample	Bond	N	R(Å)	σ^2 (10^{-3}Å^2)	ΔE_0 (eV)	R factor
**CoPc-S-COF (before tests)**	Co-N (first shell)	4	1.91±0.01	4.2±0.4	2.5±1.4	0.002
	Co-C (second shell)	8	2.99±0.01	7.1±0.5	3.5±1.0	
	Co-N (third shell)	4	3.26±0.01	7.1±0.5	3.5±1.0	
**CoPc-S-COF (after tests)**	Co-N (first shell)	4	1.92±0.02	8.7±1.1	7.9±3.6	0.003
	Co-C (second shell)	8	2.99±0.03	8.3±1.2	5.5±3.7	
	Co-N (third shell)	4	3.29±0.03	8.3±1.2	5.5±3.7	
**CoPc-O-COF (before tests)**	Co-N (first shell)	4	1.92±0.01	4.3±1.2	7.1±3.9	0.010
	Co-C (second shell)	8	3.00±0.02	7.5±1.4	4.6±2.5	
	Co-N (third shell)	4	3.27±0.03	7.5±1.4	4.6±2.5	
**CoPc-O-COF (after tests)**	Co-N (first shell)	4	1.90±0.02	7.0±1.2	4.0±2.4	0.015
	Co-C (second shell)	8	2.98±0.03	9.0±1.8	5.9±2.7	
	Co-N (third shell)	4	3.27±0.03	9.0±1.8	5.9±2.7	
**CoPc**	Co-N (first shell)	4	1.90±0.02	7.0±1.2	7.0±2.6	0.017
	Co-C (second shell)	8	2.98±0.03	9.0±1.8	6.6±3.5	
	Co-N (third shell)	4	3.27±0.03	9.0±1.8	6.6±3.5	

Supplementary Table 5. The Co content of CoPc-O-COF and CoPc-S-COF before and after stability tests.

Sample	Calculated Value (wt%)	Tested Value Before Stability Tests (wt%)	Tested Value After Stability Tests (wt%)
**CoPc-O-COF**	5.94	5.91	5.75
**CoPc-S-COF**	5.26	5.01	4.91

Supplementary Table 6. The adsorption energy (ΔG_{ads}) between O_2 and different catalytic sites in CoPc-O-COF and CoPc-S-COF.

Catalytic sites	CoPc-O-COF	CoPc-S-COF
O_2-(central Co)	-0.32 eV(attract)	-0.30 eV (attract)
O_2-(meso-N)	-0.05 eV	-0.02 eV
O_2-O	+0.62 eV (repulse)	-
O_2-S	-	+0.49 eV (repulse)
O_2-F	+0.57 eV (repulse)	+0.39 eV (repulse)

Supplementary Table 7. The condensed chemical softness (s) (|*e*|) of CoPc-S-COF and CoPc-S-COF.

<table>
<tr><th></th><th>S/O</th><th>C_a</th><th>C_b</th><th>C_c</th><th>C_d</th><th>N</th><th>Co</th></tr>
<tr><td>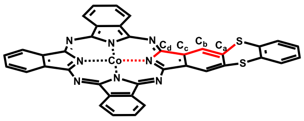CoPc-S-COF</td><td>+0.214</td><td>-0.118</td><td>+0.115</td><td>-0.051</td><td>+0.191</td><td>-0.373</td><td>+0.791</td></tr>
<tr><td>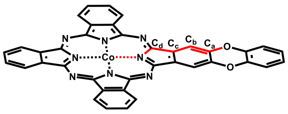CoPc-O-COF</td><td>-0.164</td><td>+0.068</td><td>+0.090</td><td>-0.024</td><td>+0.169</td><td>-0.320</td><td>+0.820</td></tr>
<tr><td rowspan="2">Difference between CoPc-S-COF and CoPc-O-COF.</td><td>0.378</td><td>-0.186</td><td>0.025</td><td>-0.030</td><td>0.022</td><td>-0.053</td><td>-0.029</td></tr>
<tr><td colspan="7">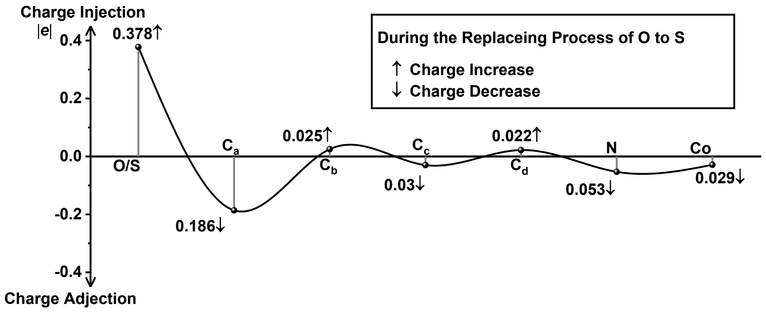</td></tr>
</table>

Supplementary Table 8. Comparison of 2e$^-$ ORR performances between CoPc-S-COF and other reported electrocatalysts in alkaline media.

	Samples	Selectivity (%)	Production capacity (mmol g_{cat}^{-1} h^{-1})	Faradaic efficiency (%)	Reference
**COF-based materials**	CoPc-S-COF	95	9500	98	This work
	MgP-DHTA-COF	96	362	91	25
	Ca-COF-318	95	453 (mmol cm^{-2} h^{-1})	91	26
	TP-TD-COF	86.2	158	-	27
	PYTA-TPEDH-COF	85.8	-	80	28
	BUCT-COF-1	54	-	-	29
	BUCT-COF-7	83.4	326.9	-	30
	Py-TD-COF	92	-	-	31
**Co-based materials**	ZnCo-ZIF-C3	99	4300	85	32
	Co-N/HPC	98	1720	92.3	33
	CoPc MDE	78	8200	88	34
	Co-N_2-C/HO	91.3	6912	-	35
**Others**	Ni-N_2O_2/C	96	5900	91	36
	C-MOF Ni-250	95	510	-	37
	Cu/Ni-HHTP	95	793	-	38
	Br-Ni MOF	86	596	-	39
	In SAs/NSBC	95	6490	-	40
	α-Fe_2O_3	96	454	-	41
	CMK3-20	90	2467	-	42
	G-COF-950	75	1287	-	43
	Fe-CNT	95	1600	-	44

3. Supplementary References.

1 Jones J. G., et al. Fluorinated iron phthalocyanine. *Inorg. Chem.* **8**, 2018-2019 (1969).

2 Ke, C. S., et al. Molecular Engineering and Design of Semiconducting Polymer Dots with Narrow-Band, Near-Infrared Emission for in Vivo Biological Imaging. *ACS Nano* **11**, 3166-3177 (2017).

3 Dirk, C. W., et al. Isolation and purification of benzene-1,2,4,5-tetrathiol. *J. Org. Chem.* **50**, 2395-2397 (1985).

4 Chen, S., et al. Identification of the Highly Active Co-N_4 Coordination Motif for Selective Oxygen Reduction to Hydrogen Peroxide. *J. Am. Chem. Soc.* **144**, 14505-14516 (2022).

5 Nørskov, J. K. , et al. Origin of the Overpotential for Oxygen Reduction at a Fuel-Cell Cathode. *J. Phys. Chem. B.* **108**, 17886-17892 (2004).

6 Kim, H.W., et al. Efficient hydrogen peroxide generation using reduced graphene oxide-based oxygen reduction electrocatalysts. *Nat. Catal.* **1**, 282-290 (2018).

7 Zhi, Q., et al. Piperazine-Linked Metalphthalocyanine Frameworks for Highly Efficient Visible-Light-Driven H_2O_2 Photosynthesis. *J. Am. Chem. Soc*, **144**, 21328-21336 (2022).

8 Zhao, Y., et al. A new local density functional for main-group thermochemistry, transition metal bonding, thermochemical kinetics, and noncovalent interactions. *J. Chem. Phys.* **125**, 194101 (2006).

9 Grimme, S., et al. A consistent and accurate *ab initio* parametrization of density functional dispersion correction (DFT-D) for the 94 elements H-Pu. *J. Chem. Phys.* **132**, 154104 (2010).

10 Wachters, A. J. H. *J. Chem. Phys.* **52**, 1033–1036 (1970).

11 Hay, P. J. Gaussian basis sets for molecular calculations. The representation of *3d* orbitals in transition-metal atoms. *J. Chem. Phys.* **66**, 4377–4384 (1977).

12 Dunning Jr. T. H., et al. In: *Modern Theoretical Chemistry, Vol. 3.* Edited by

H. F. Schaefer III. New York: Plenum, 1-28 (1977).

13 Frisch, M. J., et al. *Gaussian 16, Revision B.01*, Gaussian, Inc., Wallingford CT, 2016.

14 Gonthier, J.F., et al. π-Depletion as a criterion to predict π-stacking ability. *Chem. Commun.* **48**, 9239-9241 (2012).

15 Chai J-D., et al. Long-range corrected hybrid density functionals with damped atom-atom dispersion corrections. *Phys. Chem. Chem. Phys.* **10**, 6615-6620 (2008).

16 Chai, J.-D., et al. Systematic optimization of long-range corrected hybrid density functionals. *J. Chem. Phys.* **128**, 084106 (2008).

17 Kresse G., et al. Efficiency of ab-initio total energy calculations for metals and semiconductors using a plane-wave basis set. *Comput. Mater. Sci.* **6**, 15-50 (1996).

18 Kresse, G., et al. Efficient iterative schemes for *ab initio* total-energy calculations using a plane-wave basis set. *Phys. Rev. B: Condens. Matter Mater. Phys.* **54**, 11169-11186 (1996).

19 Blochl, P. E., et al. Projector augmented-wave method. *Phys. Rev. B* **50**, 17953-17979 (1994).

20 Perdew, J. P., et al. Generalized Gradient Approximation Made Simple. *Phys. Rev. Lett.* **78**, 1396 (1997).

21 Delley, B., An all-electron numerical method for solving the local density functional for polyatomic molecules. J. *Chem. Phys.* **92,** 508–517 (1990).

22 Delley, B. From molecules to solids with the $DMol^3$ Approach. *J. Chem. Phys.* **113**, 7756–7764 (2000).

23 Momma, K. et al. *VESTA:* a three-dimensional visualization system for electronic and structural analysis F. Izumi, *J. Appl. Crystallogr.* **41**, 653-658 (2008).

24 Carpenter, J. E. *Extension of Lewis structure concepts to open-shell and excited-state molecular species.* (PhD thesis. *University of Wisconsin*, Madison, WI, 1987).

25 Guo, Y., et al. Precise Design of Covalent Organic Frameworks for Electrocatalytic Hydrogen Peroxide Production. *Chem. Asian J.* **16**, 498-502 (2021).

25 Liu, M., et al. Construction of Atomic Metal-N_2 Sites by Interlayers of Covalent Organic Frameworks for Electrochemical H_2O_2 Synthesis *Small* **18**, e2204757 (2022).

27 Huang, S.,et al. Covalent Organic Frameworks with Molecularly Electronic Modulation as Metal-Free Electrocatalysts for Efficient Hydrogen Peroxide Production. *Small Struct.*, 2200387 (2023).

28 An, S., et al. One-Dimensional Covalent Organic Frameworks for the $2e^-$Oxygen Reduction Reaction. *Angew. Chem. Int. Ed.* **62**, e202218742 (2023).

29 Bao, R., et al. Designing Thiophene-Enriched Fully Conjugated 3D Covalent Organic Framework as Metal-Free Oxygen Reduction Catalyst for Hydrogen Fuel Cells. *Angew. Chem. Int. Ed.* **62**, e202216751 (2023).

30 Zhang, Y., et al. Multicomponent Synthesis of Imidazole-Linked Fully Conjugated 3D Covalent Organic Framework for Efficient Electrochemical Hydrogen Peroxide Production. *Angew. Chem. Int. Ed.,* **n/a**, e202314539 (2023).

31 Huang, S., *et al.* Linkage engineering in covalent organic frameworks as metal-freeoxygen reduction electrocatalysts for hydrogen peroxide production. *Appli. Catal. B.,* **340**, 123216 (2024).

32 Zhang, C., et al. Crystal Engineering Enables Cobalt-Based Metal–Organic Frameworks as High-Performance Electrocatalysts for H_2O_2 Production. *J. Am. Chem. Soc.* **145**, 7791-7799 (2023).

33 Tian, Y., et al. Edge-hosted Atomic Co-N_4 Sites on Hierarchical Porous Carbon for Highly Selective Two-electron Oxygen Reduction Reaction. *Angew. Chem. Int. Ed.* **61**, e202213296 (2022).

34 Yuan, Y., et al. Deciphering the selectivity descriptors of heterogeneous metal phthalocyanine electrocatalysts for hydrogen peroxide production *Chem. Sci.* **13**, 11260-11265 (2022).

35 Gong, H., et al. Low-Coordinated Co-N-C on Oxygenated Graphene for Efficient Electrocatalytic H_2O_2 Production*Adv. Funct. Mater.* **32**, 2106886 (2022).

36 Wang, Y., et al. High-Efficiency Oxygen Reduction to Hydrogen Peroxide Catalyzed by Nickel Single-Atom Catalysts with Tetradentate N_2O_2 Coordination in a Three-Phase Flow Cell. *Angew. Chem. Int. Ed.* **59**, 13057-13062 (2020).

37 Wu, H., et al. Activated Ni-based metal–organic framework catalyst with well-defined structure for electrosynthesis of hydrogen peroxide. *Chem. Eng. J.* **435**, 134863(2022).

38 Sun, X., et al. Dissecting π-conjugated covalent-coupling over conductive MOFs toward efficient two-electron oxygen reduction. *Appl. Catal. B: Envir.* **317**, 121706 (2022).

39 Liu, M., et al. Self-Nanocavity-Confined Halogen Anions Boosting the High Selectivity of the Two-Electron Oxygen Reduction Pathway over Ni-Based MOFs. *J. Phys. Chem. Lett.* **12**, 8706-8712 (2021).

40 Zhang, E., et al. Engineering the Local Atomic Environments of Indium Single-Atom Catalysts for Efficient Electrochemical Production of Hydrogen Peroxide. *Angew. Chem. Int. Ed.* **61**, e202117347 (2022).

41 Gao, R., et al. Engineering Facets and Oxygen Vacancies over Hematite Single Crystal for Intensified Electrocatalytic H_2O_2 Production. *Adv. Funct. Mater.* **30**, 1910539 (2020).

42 Chen, Z., et al. Development of a reactor with carbon catalysts for modular-scale, low-cost electrochemical generation of H_2O_2. *React. Chem. Eng.* **2**, 239-245 (2017).

43 Zhang, J., et al. Graphitic N in nitrogen-Doped carbon promotes hydrogen peroxide synthesis from electrocatalytic oxygen reduction. *Carbon* **163**, 154-161 (2020).

44 Jiang, K., et al. Highly selective oxygen reduction to hydrogen peroxide on transition metal single atom coordination. *Nat. Commun.* **10**, 3997 (2019).
